# Supplementary material for: Gene expression profiling reveals enhanced nutrient and drug metabolism and maturation of hiPSC-derived intestine-on-chip relative to organoids and Transwells
Source: Stem Cell Reports. 2025 Nov 13;20(12):102715. doi: 10.1016/j.stemcr.2025.102715 (PMC12744852; doi:10.1016/j.stemcr.2025.102715)
Supplement: Document S1. Figures S1 and S2, Tables S1–S3, and supplemental methods [file mmc1.pdf]

**Stem Cell Reports, Volume *20***

## Supplemental Information

# Gene expression profiling reveals enhanced nutrient and drug metabolism and maturation of hiPSC-derived intestine-on-chip relative to organoids and Transwells

**Renée Moerkens, Joram Mooiweer, Eline Smits, Marijn Berg, Aarón D. Ramírez-Sánchez, Rutger Modderman, Jens Puschhof, Cayetano Pleguezuelos-Manzano, Robert J. Barrett, Cisca Wijmenga, Iris H. Jonkers, and Sebo Withoff**

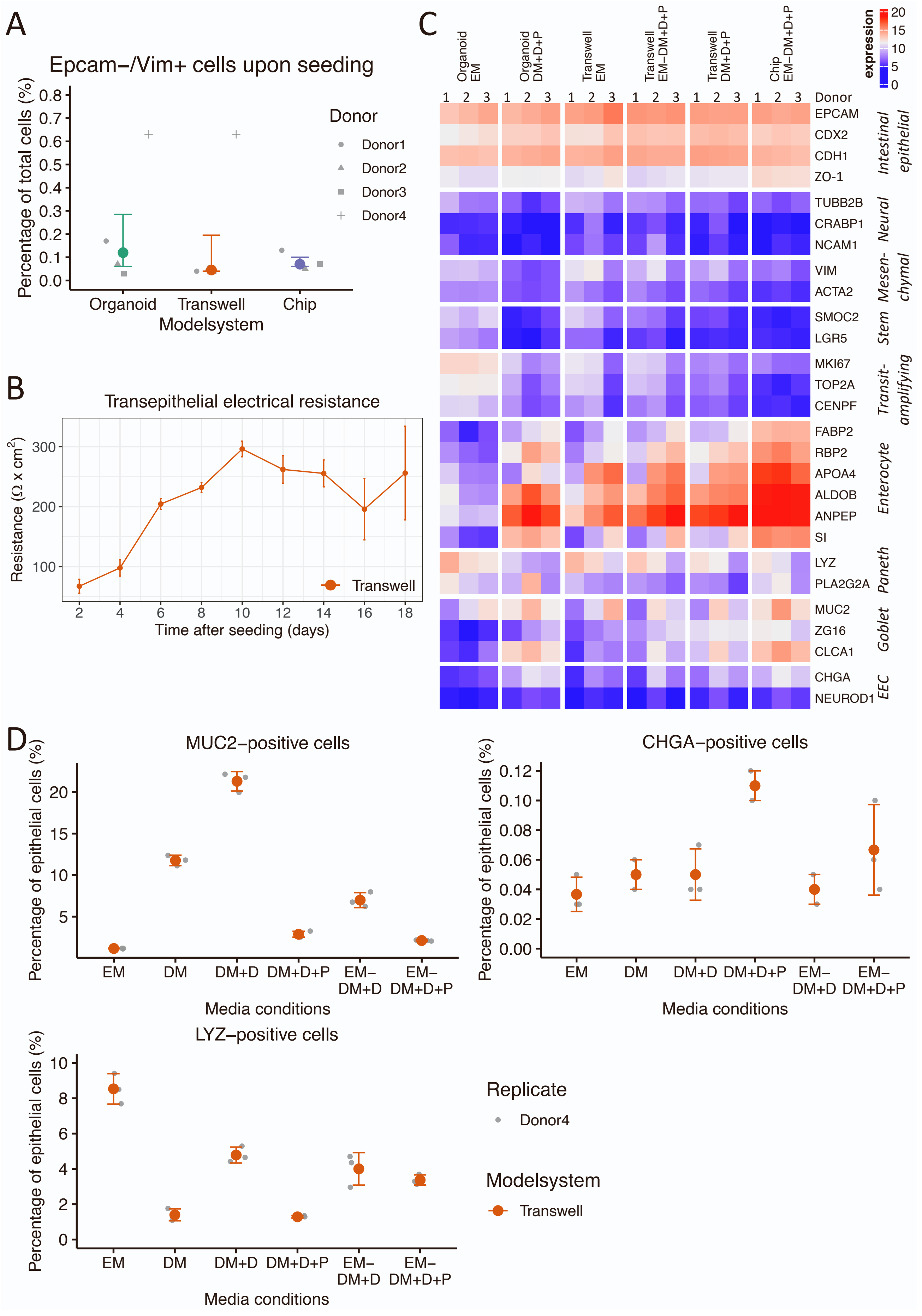
A
Epcam−/Vim+ cells upon seeding
Percentage of total cells (%)
0.8
0.7
0.6
0.5
0.4
0.3
0.2
0.1
0.0
Organoid
Transwell
Chip
Modelsystem
Donor
Donor1
Donor2
Donor3
Donor4
B
Transepithelial electrical resistance
Resistance (Ω x cm2)
300
200
100
2
4
6
8
10
12
14
16
18
Time after seeding (days)
Transwell
C
Organoid EM
Organoid DM+D+P
Transwell EM
Transwell EM−DM+D+P
Transwell DM+D+P
Chip EM−DM+D+P
expression
20
15
10
5
0
1 2 3
Donor
EPCAM
CDX2
CDH1
ZO-1
Intestinal epithelial
TUBB2B
CRABP1
NCAM1
Neural
VIM
ACTA2
Mesen-chymal
SMOC2
LGR5
Stem
MKI67
TOP2A
CENPF
Transit-amplifying
FABP2
RBP2
APOA4
ALDOB
ANPEP
SI
Enterocyte
LYZ
PLA2G2A
Paneth
MUC2
ZG16
CLCA1
Goblet
CHGA
NEUROD1
EEC
D
MUC2−positive cells
Percentage of epithelial cells (%)
20
15
10
5
EM
DM
DM+D
DM+D+P
EM−DM+D
EM−DM+D+P
Media conditions
CHGA−positive cells
Percentage of epithelial cells (%)
0.12
0.10
0.08
0.06
0.04
0.02
0.00
EM
DM
DM+D
DM+D+P
EM−DM+D
EM−DM+D+P
Media conditions
LYZ−positive cells
Percentage of epithelial cells (%)
10
8
6
4
2
0
EM
DM
DM+D
DM+D+P
EM−DM+D
EM−DM+D+P
Media conditions
Replicate
Donor4
Modelsystem
Transwell

**Figure S1. Expression of epithelial-, mesenchymal- and neural-specific markers.** (A) Flow cytometry quantification of Epcam-negative VIM-positive cells present upon seeding of the model systems, displayed as median with interquartile range of four donors. (B) Transepithelial electrical resistance (TEER) over time in Transwell systems, displayed as mean with standard deviation of three technical replicates. (C) Expression levels of genes associated with intestinal epithelial, mesenchymal and neural cells and multiple intestinal epithelial cell types in diverse model systems and medium conditions. EEC = enteroendocrine cell. Color scale represents normalized counts. (D) Flow cytometry quantification of the intra donor variation in cell type proportions, displayed as mean with standard deviation of four technical replicates.

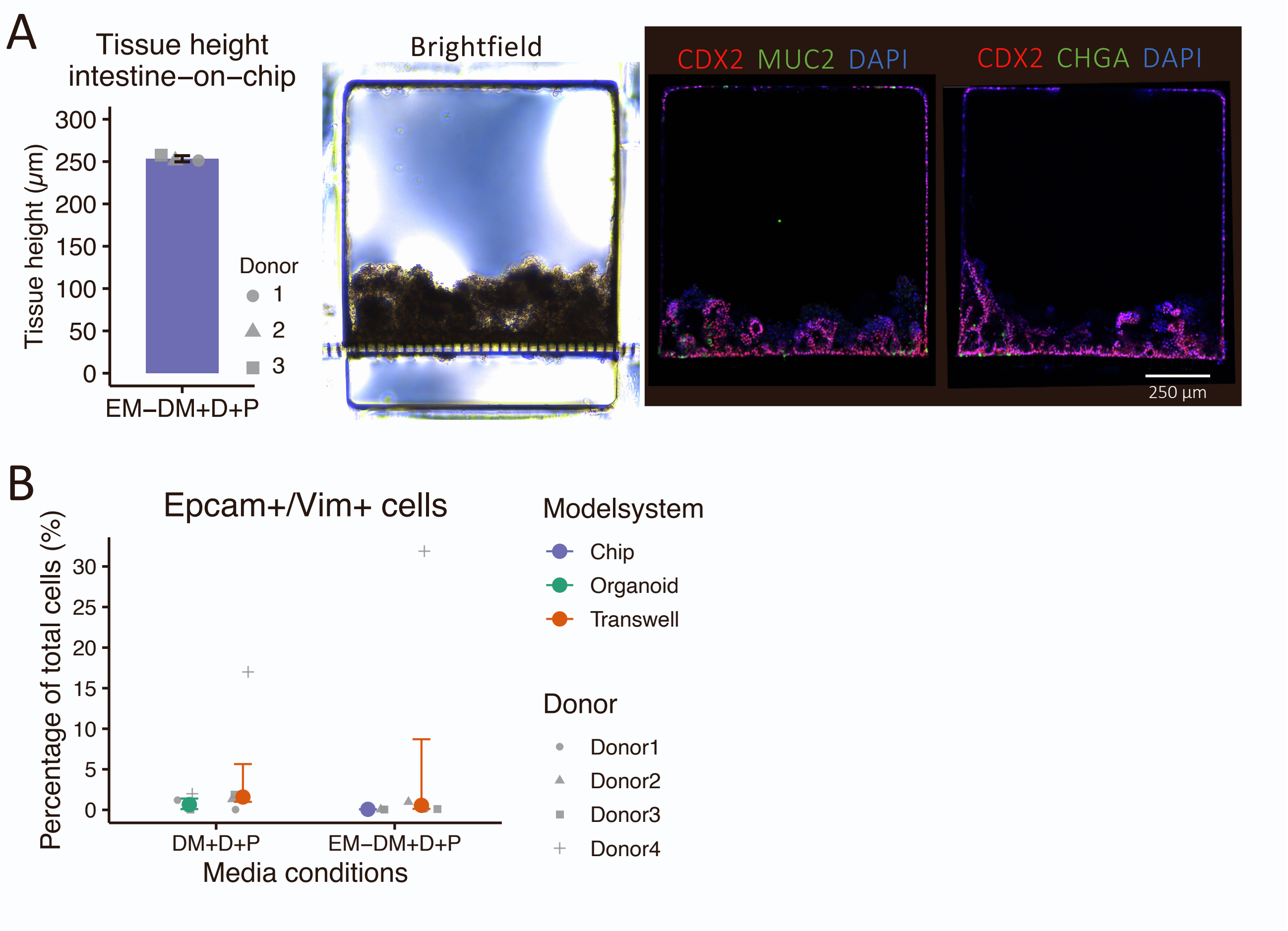


**Figure S2. Characterization of the hiPSC-derived intestine-on-chip.** (A) Tissue height quantification and representative brightfield and immunofluorescent confocal images of cross-sectional slices of intestine-on-chip systems exposed to the EM-DM+D+P condition, stained for CDX2 (intestinal epithelium), MUC2 (goblet cell) and CHGA (enteroendocrine cell). Tissue height quantification is displayed as mean with standard deviation of three donors. (B) Flow cytometry quantification of Epcam-positive VIM-positive cells in different model systems, displayed as median with interquartile range of four donors.

**Table S1. Primary antibodies used for immunofluorescent microscopy**

| Antigen | Dilution | Host/Isotype | Catalogue number | Manufacturer |
|---|---|---|---|---|
| CHGA | 1:100 | Mouse IgG1 | MA5-13096 | ThermoFisher |
| MUC2 | 1:200 | Mouse IgG1 | ab118964 | Abcam |
| LYZ | 1:200 | Mouse IgG2a | NB100-63062 | Novus Biologicals |
| MKI67 | 1:200 | Rabbit IgG | ab16667 | Abcam |
| RBP2 | 1:100 | Rabbit polyclonal | HPA035866 | Sigma Aldrich |
| ZO-1 | 1:100 | Rabbit IgG polyclonal | 61-7300 | ThermoFisher |
| CDX2 | 1:100 | Goat IgG polyclonal | AF3665-SP | R&D systems |

**Table S2. Secondary antibodies used for immunofluorescent microscopy**

| Fluorophore | Dilution | Species reactivity | Catalogue number | Manufacturer |
|---|---|---|---|---|
| Alexa Fluor 488 | 1:250 | Mouse IgG (H+L) | 715-545-150 | Jackson ImmunoResearch |
| Alexa Fluor 647 | 1:250 | Goat IgG (H+L) | A-21447 | ThermoFisher |
| Cy3 | 1:250 | Rabbit IgG (H+L) | 711-165-152 | Jackson ImmunoResearch |

**Table S3. Antibodies used for flow cytometry**

| Antigen | Fluorophore | Dilution | Catalogue number | Manufacturer |
|---|---|---|---|---|
| VIM | Alexa Fluor 594 | 1:50 | 7675S | Cell Signaling Technology |
| EPCAM/CD326 | BUV737 | 1:100 | 748397 | BD Biosciences |
| CHGA | PE | 1:1000 | ab213341 | Abcam |
| MUC2 | PerCP | 1:67 | NBP2-34757PCP | Novus Biologicals |
| LYZ | Alexa Fluor 488 | 1:200 | NB100-63062AF488 | Novus Biologicals |

## Supplemental methods

### Immunofluorescent microscopy

Cells were fixed in paraformaldehyde (PFA, 4% vol/vol DPBS, ThermoFisher #28908) for 10 min (Transwell and intestine-on-chip) or 60 min (organoids), washed and stored in DPBS at 4 °C. Cross-sectional slices of intestine-on-chip systems (200 µm thick) were generated using a vibratome (VT1000S, Leica) as described previously (Moerkens et al., 2024) and Transwell inserts were removed and divided in quarters using a scalpel and microtome blade. All samples were permeabilized in Triton X-100 (0.1% vol/vol DPBS, Sigma Aldrich #T8787) for 10-15 min (Transwell systems) or 30 min (organoids and intestine-on-chip systems) and blocked with bovine serum albumin (BSA, 3% weight/vol DPBS, Sigma Aldrich #A2153) for 1 hour. Primary antibodies diluted in BSA solution were applied overnight at 4 °C. Samples were washed twice with DPBS and secondary antibodies diluted in BSA solution were applied for 2 hours at room temperature in the dark. Samples were washed three times with DPBS and Transwell inserts and intestine-on-chip slices were mounted on a glass microscope slide in mounting medium with DAPI (Vector Laboratories #H-2000), while the Matrigel domes containing organoids in Nunc™ Lab-Tek™ Chamber Slide Systems were submerged in the mounting medium. Images were taken using a Leica SP8 CLSM confocal immunofluorescent microscope (10x and 20x objective) and analyzed using the Leica LAS X software. Primary and secondary antibodies are listed in Tables S1 and S2.

### Flow cytometry

Organoids were released from Matrigel domes and dissociated using TrypLE Select as described before. Transwell and intestine-on-chip systems were washed twice with DPBS and incubated in TrypLE Select at 37 °C. Every 10 min, detached cells were collected in a microcentrifuge tube, further incubated (5 min, 37 °C) and dissociated to a single cell suspension by gentle repeated pipetting. The single cell suspension was added to a centrifuge tube containing Advanced DMEM/F12 supplemented with FCS (10% vol/vol) and Y-27632 (10 µM) and stored on ice. Previous steps were repeated 3-4 times until the tissue was removed from the Transwell insert and top channel of the intestine-on-chip. The resulting cell suspension was passed through a 70-µm filter, centrifuged (400xg, 5 min, 4 °C) and resuspended in Advanced DMEM/F12 supplemented with FCS and Y-27632. The cells were stained using the Zombie Aqua Fixable Viability Kit (BioLegend #423101) according to the manufacturer’s instructions, fixed in PFA (4% vol/vol DPBS, ThermoFisher #28908) for 10 min and washed with DPBS. Cells were permeabilized and labelled with fluorophore-conjugated antibodies using the BD Perm/Wash buffer (BD Biosciences #554723) according to the manufacturer’s instructions. BD Horizon Brilliant Stain Buffer (BD Biosciences #563794) was included in the antibody mixture in an equal volume to the total volume of the antibodies. Flow cytometry analysis was performed using the Cytek Aurora and the Kaluza Analysis software (Beckman Coulter Life Sciences). Model system-specific unstained, single-antibody stained and fluorescence-minus-one samples were included for the gating strategy and compensation. Antibodies are listed in Table S3.

### RNA sequencing and gene expression quantification

Organoids were released from Matrigel domes as described before and lysed in Lysis/Binding Buffer from the mirVana™ miRNA Isolation Kit (ThermoFisher #AM1561). Transwell inserts were washed twice with cold DPBS and cells were lysed in Lysis/Binding Buffer. Cells were

dissociated from the intestine-on-chip as described before, washed twice with DPBS and lysed in Lysis/Binding Buffer. RNA was isolated from cell lysates using the mirVana™ miRNA Isolation Kit (ThermoFisher #AM1561) according to manufacturer's instructions. High RNA integrity (RIN > 8.3) was confirmed using the Agilent RNA ScreenTape (#5067-5576) on the Agilent TapeStation 4200. RNA library preparation and sequencing were performed in one batch at BGI Tech Solutions (Hong Kong) according to the 'DNBSEQ Eukaryotic Strand-specific mRNA library' protocol. Briefly, mRNA was isolated using oligo-dT beads and fragmented. cDNA was synthesized, sequencing adapters were ligated and PCR amplification was performed. Library quality and yield were determined and sequencing was performed using a DNBseq sequencing platform with a 150-bp paired-end (PE150) kit. Adapter sequences and low-quality reads were removed using BGI Tech Solutions' SOAPnuke software (Chen et al., 2018). The trimmed fastQ files were aligned to humanG1Kv37 reference genome (Ensembl Release 75) using Hisat (version 0.1.5) (Kim, Langmead, & Salzberg1, 2015) with default settings, and aligned reads were sorted using SAMtools (version 1.2) (Li et al., 2009). Gene-level quantification was performed using HTSeq-count HTSeq (version 0.6.1p1) (Anders, Pyl, & Huber, 2015) with --mode=union and Ensembl version 75 as gene annotation database. Quality control metrics were calculated for the raw sequencing data using FastQC (version 0.11.3) (Andrews, 2010) and for the aligned reads using Picard-tools (version 1.130). GATK tool SplitNCigarReads was used to split reads into exon segments and hard-clip sequences overhanging into the intronic regions. Variant calling was done using HaplotypeCaller in GVCF mode. All samples were then jointly genotyped by taking the gVCFs produced earlier and running GenotypeGVCFs to create a set of raw SNP and indel calls per chromosome (McKenna, 2010).

**Distance matrix analysis**

Genes representing the epithelial compartment of the human small intestine were determined using the single-cell RNA sequencing data of the Gut Cell Atlas (Elmentaite et al., 2021, 2020). The data was subsetted to exclude inflammatory bowel disease-related samples (Diagnosis = 'Pediatric Crohn Disease') and select for cells of the small intestine (Region = 'SmallInt'). DEGs were identified using the FindMarkers function of Seurat (version 5.0.2), contrasting the intestinal epithelial cells (category = 'Epithelial') to all other cellular compartments (mesenchymal, neural, endothelial and immune). 'Intestinal epithelial marker genes' were filtered as having adjusted p-value < 0.05 and falling in the fourth quantile of log2FoldChange (corresponding to log2FoldChange ≥ 2.7), resulting in 993 genes. To perform the distance matrix analysis, the Gut Cell Atlas data (Elmentaite et al., 2021, 2020) was subsetted for small intestinal epithelial cells using the same parameters as indicated above and additionally selecting for epithelial cells (category = 'Epithelial') and excluding one donor (Sample.name = 'A32 (411C)') based on the aberrant Paneth cell ratio of 50% from all epithelial cells in an ileal sample. Subsequently, pseudo-bulk data was generated by aggregating counts per age group (variable 'Age_group') and donor (variable 'Sample.name') using the Seurat function AggregateExpresssion. Raw counts of shared genes between the hiPSC-derived intestinal data (organoids, Transwell and intestine-on-chip system) described in this manuscript, the Gut Cell Atlas data and the RNA-sequencing data of the epithelial layer of human duodenal biopsies from healthy adults described by Ramírez-Sánchez et al. (Ramírez-Sánchez et al., 2024) were merged in one count matrix. The euclidean distance between the samples was determined using the VST-normalized expression (DESeq2) of

intestinal epithelial marker genes present in the count matrix using the dist() function in R and scaled per column for the visualization.

**Transepithelial electrical resistance in Transwells**

Transepithelial electrical resistance (TEER) was measured every second day in Transwell systems with the Millicell ERS-2 Voltohmmeter (Millipore, #MERS00002). For each timepoint, the average resistance from three positions per insert was calculated, and the resistance of an empty insert was subtracted to correct for background. Subsequently, the unit area resistance ($\Omega x cm^2$) was calculated by multiplying the resistance ($\Omega$) by the effective membrane area of the inserts (1.12 $cm^2$).

**Tissue height quantification in intestine-on-chip**

Tissue height was calculated from cross-sectional chip slices using Fiji-2 by averaging the shortest and longest distances from the tissue surface in the top channel to the chip membrane and converting to height using the scale bar. For each donor, the average was calculated of four slices from different positions along the length of the chip.

## Supplemental references


Anders, S., Pyl, P. T., & Huber, W. (2015). HTSeq-A Python framework to work with high-throughput sequencing data. *Bioinformatics*, *31*(2), 166–169. https://doi.org/10.1093/bioinformatics/btu638

Andrews, S. (2010). FastQC a Quality Control Tool for High Throughput Sequence Data.

Chen, Y., Chen, Y., Shi, C., Huang, Z., Zhang, Y., Li, S., … Chen, Q. (2018). SOAPnuke: A MapReduce acceleration-supported software for integrated quality control and preprocessing of high-throughput sequencing data. *GigaScience*, *7*(1), 1–6. https://doi.org/10.1093/gigascience/gix120

Elmentaite, R., Kumasaka, N., Roberts, K., Fleming, A., Dann, E., King, H. W., … Teichmann, S. A. (2021). Cells of the human intestinal tract mapped across space and time. *Nature*, *597*(7875), 250–255. https://doi.org/10.1038/s41586-021-03852-1

Elmentaite, R., Ross, A. D. B., Roberts, K., James, K. R., Ortmann, D., Gomes, T., … Zilbauer, M. (2020). Single-Cell Sequencing of Developing Human Gut Reveals Transcriptional Links to Childhood Crohn's Disease. *Developmental Cell*, *55*(6), 771-783.e5. https://doi.org/10.1016/j.devcel.2020.11.010

Kim, D., Langmead, B., & Salzberg1, S. L. (2015). HISAT: a fast spliced aligner with low memory requirements Daehwan HHS Public Access. *Nature Methods*, *12*(4), 357–360. https://doi.org/10.1038/nmeth.3317.HISAT

Li, H., Handsaker, B., Wysoker, A., Fennell, T., Ruan, J., Homer, N., … Durbin, R. (2009). The Sequence Alignment/Map format and SAMtools. *Bioinformatics*, *25*(16), 2078–2079. https://doi.org/10.1093/bioinformatics/btp352

McKenna, A. (2010). The Genome Analysis Toolkit: a MapReduce framework for analyzing next-generation DNA sequencing data.

Moerkens, R., Mooiweer, J., Ramírez-Sánchez, A. D., Oelen, R., Franke, L., Wijmenga, C., … Withoff, S. (2024). An iPSC-derived small intestine-on-chip with self-organizing epithelial, mesenchymal and neural cells. *Cell Reports*, *43*(7), 114247. https://doi.org/https://doi.org/10.1016/j.celrep.2024.114247

Ramírez-Sánchez, A. D., Zühlke, S., Aguirre-Gamboa, R., Vochteloo, M., Franke, L., Lundin, K. E. A., … Jonkers, I. H. (2024). Gene expression and eQTL analysis reflect the heterogeneity in the inflammatory status of the duodenal epithelial lining in coeliac disease. *BioRxiv*. https://doi.org/https://doi.org/10.1101/2024.02.29.582756
