# Supplementary material for: Gene expression profiling reveals enhanced nutrient and drug metabolism and maturation of hiPSC-derived intestine-on-chip relative to organoids and Transwells
Source: Stem Cell Reports. 2025 Nov 13;20(12):102715. doi: 10.1016/j.stemcr.2025.102715 (PMC12744852; doi:10.1016/j.stemcr.2025.102715)
Supplement: Document S2. Article plus supplemental information [file mmc3.pdf]

STEM CELL REPORTS
Article
OPEN ACCESS

Gene expression profiling reveals enhanced nutrient and drug metabolism and maturation of hiPSC-derived intestine-on-chip relative to organoids and Transwells

Renée Moerkens,[1,6] Joram Mooiweer,[1,6] Eline Smits,[1,7] Marijn Berg,[1,7] Aarón D. Ramírez-Sánchez,[1] Rutger Modderman,[1] Jens Puschhof,[2] Cayetano Pleguezuelos-Manzano,[3] Robert J. Barrett,[4,5] Cisca Wijmenga,[1] Iris H. Jonkers,[1,8] and Sebo Withoff[1,8,9,*]

[1]Department of Genetics, University of Groningen, University Medical Center Groningen, 9700 RB Groningen, the Netherlands
[2]Epithelium Microbiome Interaction Laboratory, Microbiome and Cancer Division, German Cancer Research Center (DKFZ), Im Neuenheimer Feld 280, 69120 Heidelberg, Germany
[3]Hubrecht Institute, Royal Netherlands Academy of Arts and Sciences (KNAW) and UMC Utrecht, 3584 CT Utrecht, the Netherlands
[4]Board of Governors Regenerative Medicine Institute, Cedars-Sinai Medical Center, Los Angeles, CA 90048, USA
[5]F. Widjaja Foundation Inflammatory Bowel Disease Institute, Cedars-Sinai Medical Center, Los Angeles, CA 90048, USA
[6]These authors contributed equally
[7]These authors contributed equally
[8]Senior author
[9]Lead contact
*Correspondence: s.withoff@umcg.nl
https://doi.org/10.1016/j.stemcr.2025.102715

SUMMARY

The human intestinal epithelial barrier is shaped by biological and biomechanical cues, including growth factor gradients and fluid flow. While these factors are known to affect adult stem cell (ASC)-derived intestinal epithelial cells *in vitro*, their impact on human induced pluripotent stem cell (hiPSC)-derived cells is largely unexplored. Here, we compare the cellular composition and gene expression profiles of hiPSC-derived intestinal epithelial cells exposed to various medium compositions and cultured as organoids, in Transwell and microfluidic intestine-on-chip systems. Modulating key signaling pathways (WNT, NOTCH, bone morphogenetic protein [BMP], and mitogen-activated protein kinase [MAPK]) influenced the presence of dividing, absorptive, and secretory epithelial lineages. Upon differentiation, intestinal epithelial cells expressed genes encoding digestive enzymes, nutrient transporters, and drug-metabolizing enzymes. Notably, these pathways were most enhanced in the intestine-on-chip system, along with an expression profile that suggests a more mature state. These findings highlight the potential of hiPSC-derived intestinal cells to model important intestinal functions and guide the selection of optimal culture conditions for specific applications.

INTRODUCTION

The human intestinal epithelial barrier comprises diverse proliferative, secretory, and absorptive cell types that facilitate nutrient digestion and absorption and protect against harmful environmental agents. These processes vary between individuals due to genetic differences (Tracy et al., 2016; Walther et al., 2019), highlighting the importance of personalized intestinal model systems to study digestion, drug metabolism, and drug sensitivity. Human intestinal epithelial cells can be derived from adult stem cells (ASCs) in intestinal tissue (Sato et al., 2011) or from human induced pluripotent stem cells (hiPSCs) (Spence et al., 2011; Takahashi et al., 2007). In contrast to ASCs, which are acquired through invasive procedures, hiPSCs can be generated from accessible sources such as blood, skin, or urine (Raab et al., 2014). Moreover, the pluripotent character of hiPSCs allows differentiation of diverse autologous cell types and tissues for studying cellular interactions.

The diversity and functioning of intestinal epithelial cell types *in vitro* are influenced by biological and biomechanical cues that emulate intestinal processes, such as growth factor gradients, tissue elasticity, and fluid flow (Kasendra et al., 2020; Mitrofanova et al., 2024; Wang et al., 2017). The impact of these culture conditions has been extensively studied in ASC-derived intestinal epithelial cells. In these cells, lineage specification is well established via modulation of WNT, bone morphogenetic protein (BMP), NOTCH, and mitogen-activated protein kinase (MAPK) signaling pathways, reflecting the signaling patterns along the crypt-villus axis in the human intestine (Beumer and Clevers, 2021). Conversely, hiPSC-derived intestinal tissues often depend on spontaneous lineage induction achieved through elongated periods of culturing (Spence et al., 2011) or maturation *in vivo* in mice (Finkbeiner et al., 2015; Watson et al., 2014), limiting control over the cell type composition. Previous studies using ASC-derived intestinal tissues in microfluidic intestine-on-chip systems have demonstrated the value of continuous fluid flow and shear stress for the induction of villus-like folds and the expression of gene families that regulate nutrient and drug metabolism and absorption, i.e., cytochrome P450, solute carrier (SLC) transporters,

© 2025 The Authors.
Published by Elsevier Inc. on behalf of International Society for Stem Cell Research.
This is an open access article under the CC BY license (http://creativecommons.org/licenses/by/4.0/).

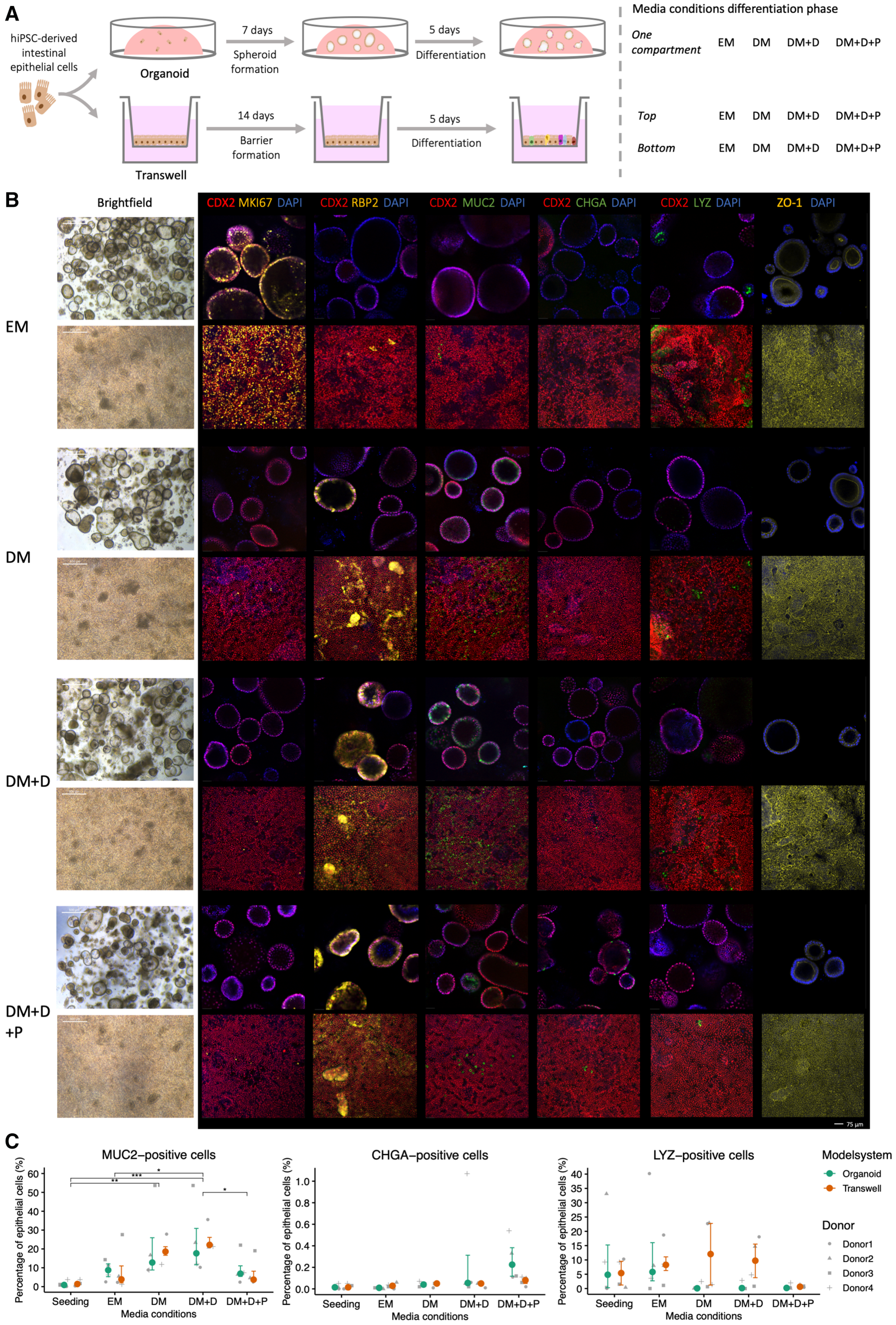

Figure 1. Induction of epithelial lineages in hiPSC-derived intestinal organoids and monolayers in a Transwell system
(A) Schematic of the experimental setup. hiPSC, human induced pluripotent stem cell; EM, expansion medium; DM, differentiation medium; DM + D, DM + DAPT; DM + D + P, DM + DAPT + PD0325901.

(legend continued on next page)

and digestive enzymes (Kasendra et al., 2020; Sugimoto et al., 2021). Moreover, model systems with two compartments, such as Transwell and intestine-on-chip systems, allow easy access to the apical and basolateral sides of the intestinal epithelial barrier. This facilitates the application of growth factor gradients to increase epithelial diversity and enables the study of barrier function or translocation of compounds, which is challenging in organoids due to their closed lumen and heterogeneity in size and shape.

In this study, we investigate the effect of diverse culture conditions on the cell type composition, gene expression profiles, and maturation status of hiPSC-derived intestinal epithelial cells. We compared three widely used model systems: intestinal organoids, Transwell systems, and a commercially available intestine-on-chip system, along with different medium compositions to enrich for specific epithelial cell types. Hereby, we provide insight into the relevant conditions and systems for modeling specific intestinal functions using hiPSC-derived intestinal epithelial cells.

RESULTS

WNT, BMP, NOTCH, and MAPK regulators induce specific epithelial lineages in hiPSC-derived intestinal epithelial organoids and monolayers in a Transwell system

In ASC-derived intestinal tissues, regulators of the WNT, BMP, NOTCH, and MAPK pathways efficiently enrich for specific epithelial cell types, providing methods to control the epithelial composition *in vitro* (Beumer and Clevers, 2021; Pleguezuelos-Manzano et al., 2020). Here, we investigated the contribution of these pathways to epithelial fate specification in hiPSC-derived intestinal epithelial organoids and monolayers in a Transwell system.

To generate a relatively pure population of intestinal epithelial cells, we subjected intestinal organoids from three hiPSC lines to immunomagnetic epithelial cell adhesion molecule (EPCAM) selection and multiple subsequent passaging steps during a 4- to 5-week expansion phase, reducing the EPCAM-negative cells that co-developed during the differentiation to less than 0.7% (Figure S1A). After seeding these intestinal epithelial cells in Matrigel domes or on Transwell inserts, we exposed them to an expansion medium (EM), allowing the formation of sufficiently sized spheroids (7 days) or a polarized epithelial barrier (14 days), respectively (Figures 1A and S1B). The subsequent differentiation phase consisted of a 5-day exposure to various medium compositions that have been described to control the induction of different epithelial lineages (Figure 1A) (Beumer and Clevers, 2021; Pleguezuelos-Manzano et al., 2020). In ASC-based tissues, EM (including WNT pathway activators and BMP pathway inhibitors) induces expansion of proliferating cells, while differentiation medium (DM; excluding WNT activators and BMP inhibitors) yields a general differentiation-inducing medium. To induce differentiation toward secretory lineages, we added a NOTCH inhibitor (DAPT) to DM (DM + D). To enrich for enteroendocrine cells, we added an MAPK inhibitor (PD0325901) to DM + D (DM + D + P).

Proliferative epithelial cells (MKI67-positive), likely stem cells and transit-amplifying cells, were abundant in the EM condition but scarce in the different DM conditions (Figure 1B). Transcriptional analysis comparing the EM and DM + D + P conditions also revealed that exposure to DM + D + P decreased stem cell markers *LGR5* and *SMOC2* and cell-cycle-associated genes *MKI67*, *TOP2A*, *PCNA*, and *CENPF* (Figure S1C). Immunofluorescence analysis showed that all three types of DM increased the number of RPB2-positive enterocytes, whereas very few were observed in the EM condition (Figure 1B). MUC2-positive epithelial cells, annotated as goblet cells, were present in the EM condition (organoid: 8.6% [SD 5.3], Transwell: 9.1% [SD 12.5]) but were induced to higher levels in DM (organoid: 22.0% [SD 21.4], Transwell: 19.2% [SD 6.6]) and DM + D (organoid: 24.8% [SD 20.1], Transwell: 25.2% [SD 7.0]), which is formulated to induce secretory progenitors (Figures 1B and 1C). The induction of MUC2-positive goblet cells was abrogated by additional MAPK inhibition in DM + D + P (organoid: 9.5% [SD 8.6], Transwell: 7.2% [SD 7.9]), in accordance with the literature describing that goblet cells arise from highly proliferating secretory progenitors (Figures 1B and 1C) (Beumer and Clevers, 2021). In contrast, Chromogranin (CHGA)-positive enteroendocrine cells arise from slow-dividing secretory progenitors (Beumer and Clevers, 2021) and showed a tendency toward induction by the inhibition of the MAPK pathway (DM + D + P), although their numbers remained relatively low (organoid: 0.3% [SD 0.2%], Transwell: 0.07% [SD 0.04]) (Figures 1B and 1C). All these epithelial cell types showed similar trends in organoids and Transwell systems, except for Lysozyme (LYZ)-positive cells (Figures 1B and 1C). Based on the absence of defensin gene expression, these cells

(B) Representative bright-field and immunofluorescent confocal images of organoids (upper lane) and monolayers on Transwell inserts (lower lane) stained for CDX2 (intestinal epithelium), MKI67 (proliferative cell), RBP2 (enterocyte), MUC2 (goblet cell), CHGA (enteroendocrine cell), LYZ (Paneth cell), and ZO-1 (tight junctions).
(C) Flow cytometry quantification of cell type proportions, displayed as median with interquartile range of four donors. p value $\leq$ 0.05 (*), 0.01 (**).

were assumed to resemble the Paneth-like cell population previously identified by our group in a hiPSC-intestine-on-chip (Moerkens et al., 2024). While LYZ-positive Paneth-like cells were present in all conditions except DM + D + P in Transwell systems, they were only detected in the EM condition in organoids, suggesting a differential sensitivity to the microenvironments of the organoids and Transwell systems (Figures 1B and 1C). The induction of cell type-specific markers, as determined on protein level (Figures 1B and 1C), corresponded with differences in gene expression levels of these and other markers characteristic of enterocytes, goblet cells, enteroendocrine cells, and Paneth cells measured by RNA sequencing for the EM and DM + D + P conditions (Figure S1C). Overall, the intra-donor variation of epithelial cell types was low (Figure S1D). The network of tight junctions between epithelial cells, as measured by the expression and localization of ZO-1, remained intact in all medium conditions regardless of the induction of different epithelial cell types (Figures 1B and S1C). Intestinal organoids were organized with the apical side projected toward the lumen, as shown by the localization of ZO-1 (Figure 1B).

Overall, in both organoids and Transwell systems, the numbers of proliferating epithelial cells were reduced and the numbers of differentiated cells were increased upon exposure to different types of DM. The removal of WNT activators and BMP inhibitors in DM was sufficient to induce goblet cells and enterocytes, while the addition of NOTCH and MAPK inhibitors (DM + D + P) was necessary to induce low levels of enteroendocrine cells. LYZ-positive Paneth-like cells were differentially induced in organoids and Transwell systems.

Growth factor gradients in Transwell systems balance proliferating and differentiating epithelial cell types

Next, we assessed whether a local inhibition and activation of the WNT, BMP, NOTCH, and MAPK pathways could sustain both dividing and differentiated epithelial cell types. We exposed the Transwell system to EM in the lower compartment and DM + D or DM + D + P in the upper compartment to mimic the growth factor gradients along the crypt-villus axis in the human intestine (Figure 2A).

In both gradient conditions, proliferating epithelial cells were sustained while differentiated epithelial cell types were induced (Figures 2B and 2C). MKI67-positive proliferating cells were abundant in the gradient conditions (Figure 2B), while they were almost entirely absent in non-gradient DM conditions (Figure 1B). More RBP2-positive enterocytes were observed in both gradient conditions relative to the EM condition (Figure 2B). In concordance with observations in DM + D and DM + D + P, MUC2-positive goblet cells were elevated in EM-DM + D (13.1% [SD 7.1]), and CHGA-positive enteroendocrine cells appeared to be induced in EM-DM + D + P (0.06% [SD 0.03]) when compared to the EM condition (Figures 2B and 2C). The levels of LYZ-positive Paneth-like cells in EM-DM + D were similar to those seen in the EM condition but slightly reduced in EM-DM + D + P (Figures 2B and 2C). A network of tight junction protein ZO-1 was observed in both gradient conditions, suggesting the preservation of barrier integrity (Figure 2B). We further confirmed the more balanced expression of dividing and differentiated epithelial cell types upon exposure to EM-DM + D + P relative to EM and DM + D + P on gene expression level (Figure S1C).

Strikingly, donor 3 displayed lower levels of stem cell and cell-cycle genes and LYZ-positive Paneth-like cells and higher levels of markers corresponding to differentiated epithelial cell types (e.g., MUC2) in all medium conditions and in the EM condition in particular (Figures 2C and S1C). We noticed that these observations correlated with a lower expression of Vimentin (VIM) in donor 3 compared to donors 1 and 2 (Figure S1C). VIM is a marker characteristic of mesenchymal cells that was expressed at low levels regardless of the limited number of EPCAM-negative and VIM-positive mesenchymal cells (below 0.7% upon seeding) (Figures S1A and S1C). It is possible that higher numbers of mesenchymal (VIM-positive) cells in donors 1 and 2 support the undifferentiated and Paneth cell state of hiPSC-derived intestinal epithelial cells and hereby prevent spontaneous differentiation.

In conclusion, gradient conditions in Transwell systems efficiently induced differentiated epithelial cell types, including enterocytes and goblet cells, while preserving proliferating epithelial cells.

Transcriptomic analysis confirms enrichment of enterocyte- and enteroendocrine-specific functions upon exposure to DM + D + P

To obtain deeper insight into the biological processes corresponding to the changing epithelial compositions, we compared the transcriptomic profiles of hiPSC-derived intestinal epithelial cells in different medium conditions using RNA sequencing. We used organoids grown in the EM and DM + D + P conditions for this analysis because we observed a diverse epithelial composition, including goblet cells, enteroendocrine cells, and enterocytes, in the latter condition. We clustered differentially expressed genes (DEGs) between the conditions into two groups depending on whether they were upregulated in the EM or DM + D + P condition and then identified biological processes by pathway enrichment analysis (Figures 3A–3C; Data S1). DEGs upregulated in the EM condition were involved in processes related to DNA replication, translation, and cell division (Figures 3B and 3C), consistent with the high abundance of proliferating epithelial cells in the EM condition relative to DM + D + P (Figure 1B).

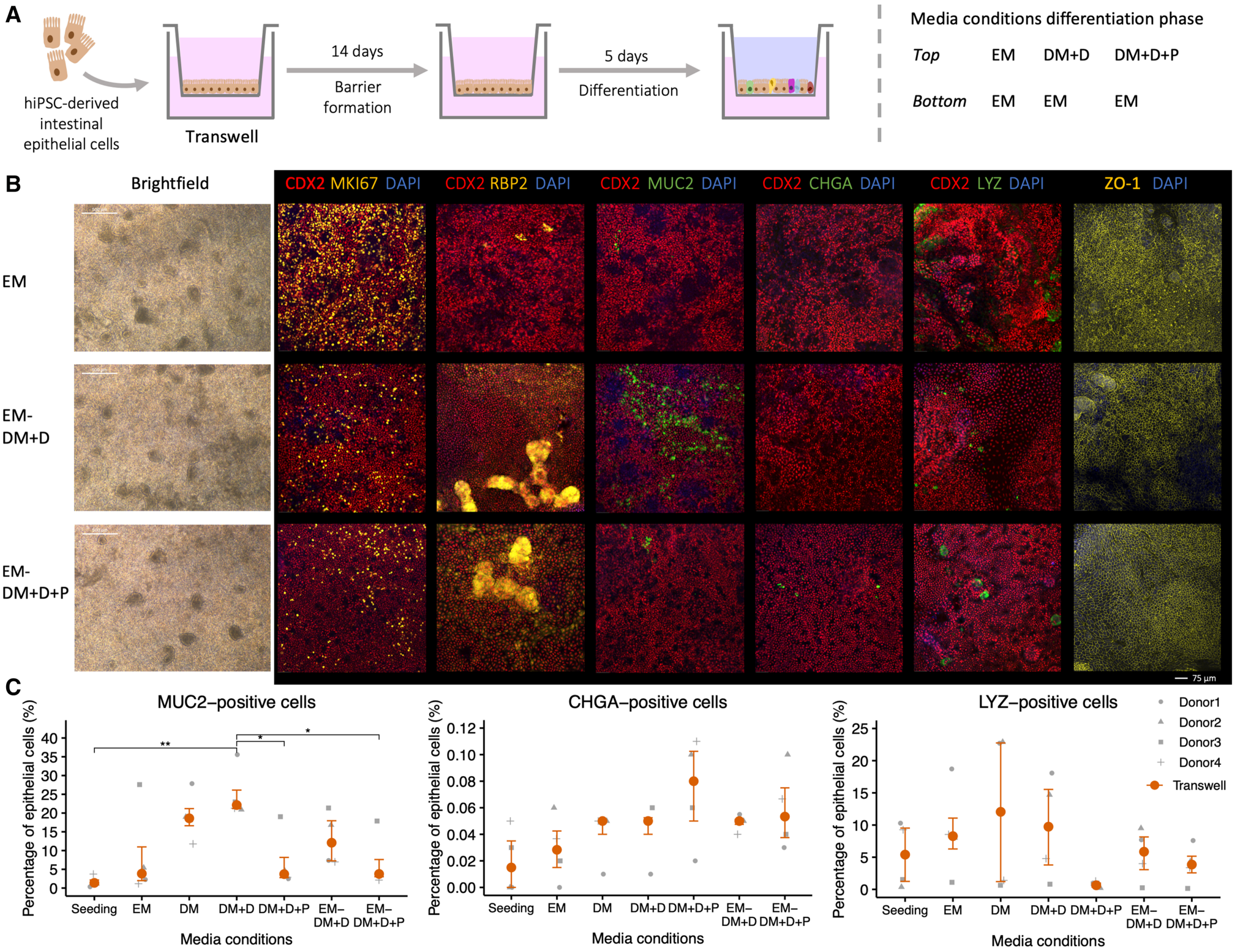

Figure 2. Balancing proliferating and differentiating epithelial cell types using growth factor gradients in Transwell systems

(A) Schematic of the experimental setup. hiPSC, human induced pluripotent stem cell; EM, expansion medium; DM, differentiation medium; DM + D, DM + DAPT; DM + D + P, DM + DAPT + PD0325901.

(B) Representative bright-field and immunofluorescent confocal images of monolayers on Transwell inserts stained for CDX2 (intestinal epithelium), MKI67 (proliferative cell), RBP2 (enterocyte), MUC2 (goblet cell), CHGA (enteroendocrine cell), LYZ (Paneth cell), and ZO-1 (tight junctions).

(C) Flow cytometry quantification of cell type proportions, displayed as median with interquartile range of four donors. p value ≤ 0.05 (*). The data of the EM, DM, DM + D, and DM + D + P conditions in (B) and (C) were repeated from Figure 1 for comparison.

In the DM + D + P condition, we observed the upregulation of genes associated with nutrient metabolism and transport, xenobiotic metabolism, and hormone responses, reflecting the induction of enterocytes and enteroendocrine cells (Figure 3B). Specifically, we observed the induction of digestive enzymes (e.g., *ANPEP*, *SI*, *LCT*, *PEPD*, *LIPA*, and *ALDOB*) and nutrient transporters (e.g., *APOB*, *APOA2*, *SLC2A2*, *SLC15A1*, *SLC5A9*, and *SLC7A9*) involved in protein, carbohydrate, and lipid metabolism characteristic of the human small intestine (Burclaff et al., 2022; Wang et al., 2020) (Figure 3C). Many of these processes were related to lipid metabolism and synthesis of carboxylic and organic acids (Figure 3B), common by-products of small intestinal digestion (Jang et al., 2018). We also observed increased expression of drug-metabolizing enzymes and transporters (e.g., *CYP3A4*, *CYP3A5*, *CY1A1*, *CYP2C9*, *ABCC2*, *UGT2A3*, and *MAOB*), comprising multiple genes associated with first-pass metabolism of xenobiotic compounds in the intestine (Fritz et al., 2019; Murata et al., 2023) (Figures 3B and 3C). *CYP3A4*, one of the most well-studied drug-metabolizing enzymes and responsible for the conversion of more than half of all drugs (Guengerich, 1999), showed the highest induction of all the cytochrome P450 members identified in DM + D + P

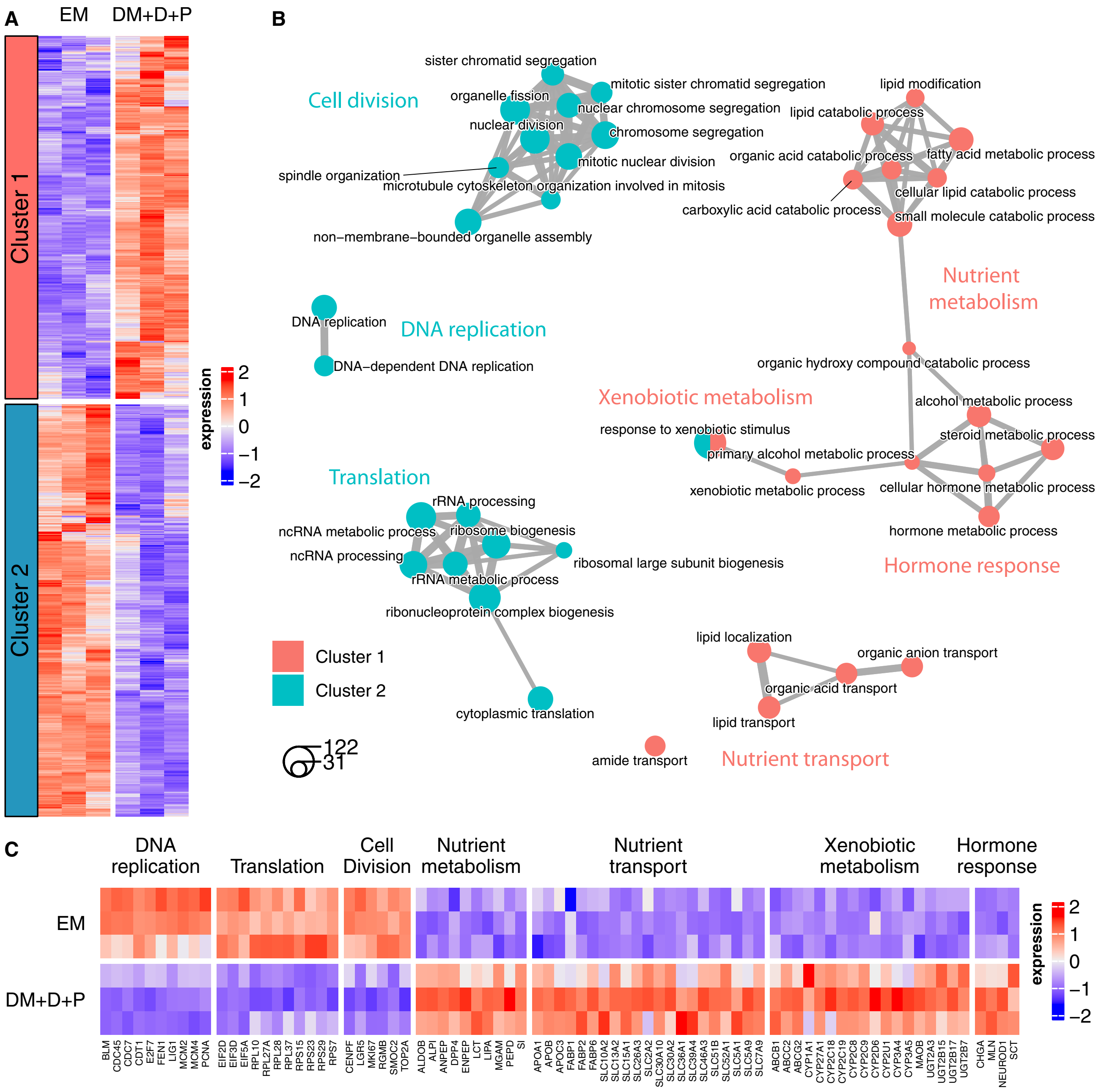

Figure 3. Enrichment of enterocyte- and enteroendocrine-specific gene expression profiles in intestinal organoids exposed to DM + D + P

(A) Heatmap of average expression of DEGs between medium conditions, clustered by expression profile. Color represents row *Z* score. Columns correspond to donors (left = 1, middle = 2, and right = 3).

(B) Network of enriched biological processes, including top 20 with lowest adjusted *p* value per cluster. Node size reflects the number of DEGs per biological process, and edge thickness indicates the number of shared DEGs.

(C) Heatmap of average expression of DEGs selected from enriched processes described in (B). Color represents column *Z* score. Rows correspond to donors (top = 1, middle = 2, and bottom = 3).

relative to EM (Figure 3C). In addition, we observed an induction of (pro-)hormones involved in gut permeability and immune activation (*CHGA*) (Eissa et al., 2018; Muntjewerff et al., 2021), gastric motility (*MLN*) (Ohno et al., 2010), and pH regulation (*SCT*) (Afroze et al., 2013) in the human intestine (Figure 3C).

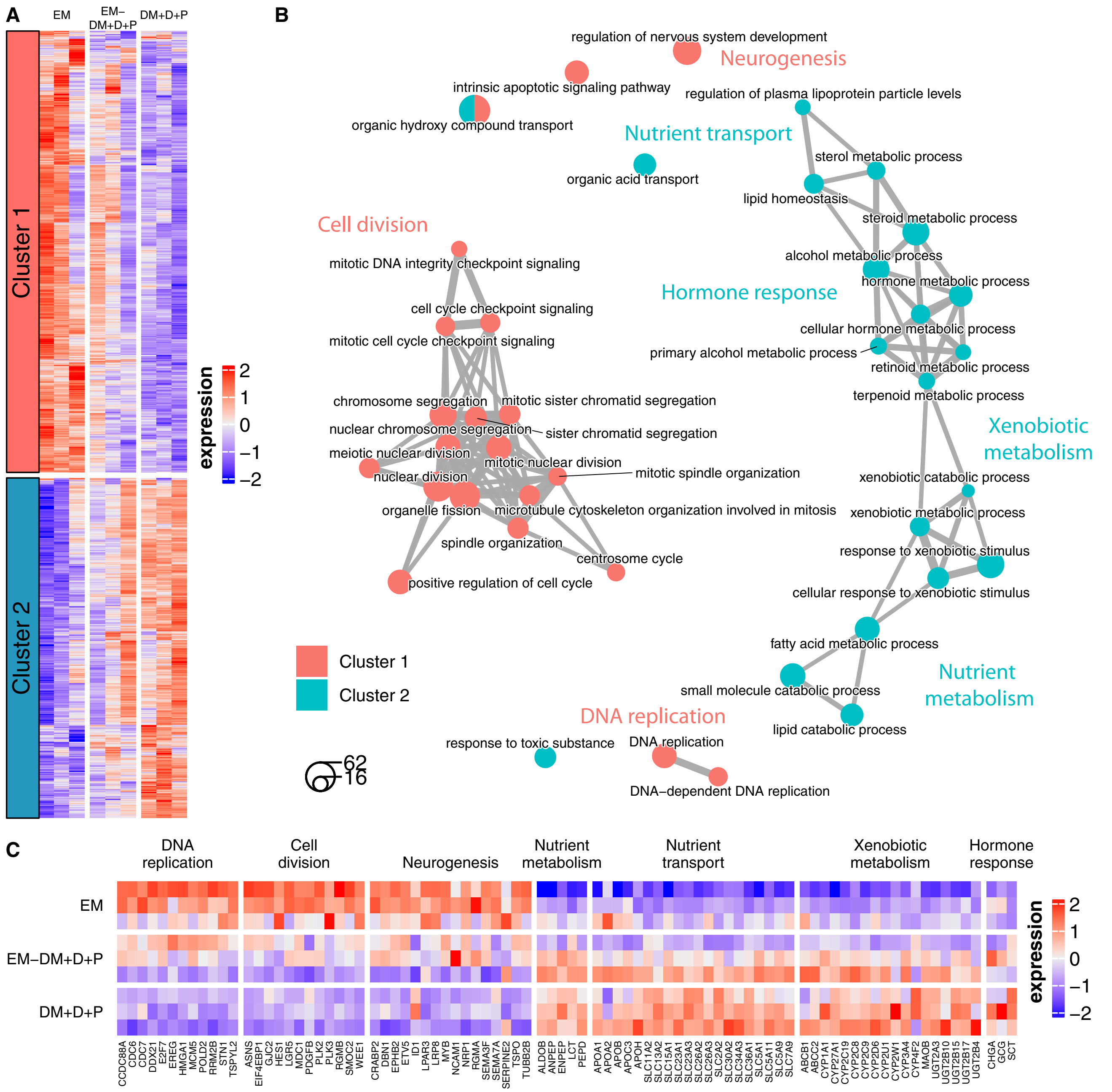

Figure 4. Balancing proliferation- and differentiation-specific gene expression profiles in Transwell systems exposed to EM-DM + D + P

(A) Heatmap of average expression of the DEGs between medium conditions, clustered by expression profile. Color represents row *Z* score. Columns correspond to donors (left = 1, middle = 2, and right = 3).

(B) Network of enriched biological processes, including top 20 with lowest adjusted *p* value per cluster. Node size reflects the number of DEGs per biological process, and edge thickness indicates the number of shared DEGs.

(C) Heatmap of average expression of DEGs selected from enriched processes described in (B). Color represents column *Z* score. Rows correspond to donors (top = 1, middle = 2, and bottom = 3).

The same analysis was performed using intestinal epithelial cells grown in Transwell systems and exposed to the EM, DM + D + P, and gradient EM-DM + D + P conditions (Figures 4A–4C; Data S1). The genes and biological processes induced by the different conditions were relatively consistent between organoids and Transwell systems and

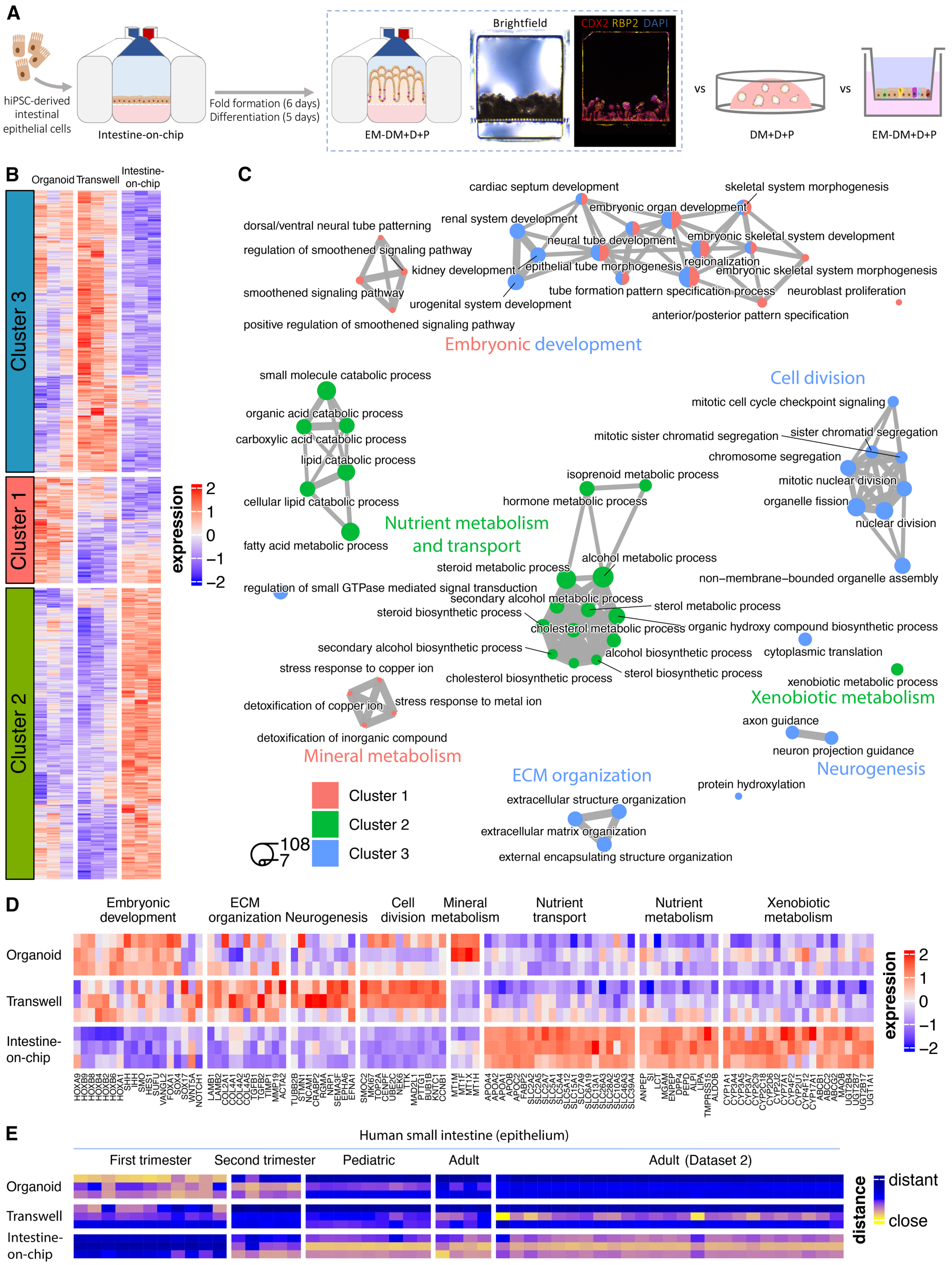

(legend on next page)

included the induction of DNA replication and cell division in the EM condition and nutrient and xenobiotic metabolism and transport as well as hormone responses in the DM + D + P condition (Figures 4B and 4C). The EM-DM + D + P condition in Transwell systems induced gene expression profiles intermediate between the profiles induced in the EM and DM + D + P conditions, in accordance with the presence of proliferating and mature epithelial cell types we observed before (Figures 2B, 2C, and 4A). Interestingly, in contrast to organoids, pathways and genes related to neurogenesis were upregulated (e.g., *TUBB2B*, *NCAM1*, *CRABP2*, *ETV5*, *NRP1*, and *RGMA*) in the EM condition in Transwell systems, which might correspond to previous observations of neuron development in a hiPSC-derived intestine-on-chip exposed to the same medium (Moerkens et al., 2024) (Figures 4B and 4C). Regardless of the limited number of EPCAM-negative cells, these mesenchymal and neural cell types might still be present at low levels and be specifically amplified in Transwell systems.

Altogether, these genes and processes are signatures of the enrichment of transit-amplifying cells and intestinal stem cells in the EM condition and the enrichment of mature enterocytes and enteroendocrine cells in the DM + D + P condition in both intestinal organoids and Transwell systems. In contrast to organoids, Transwell systems allow for gradient conditions that balance proliferative and mature epithelial cell types and processes, thereby providing a more physiological and potentially sustainable condition.

Enrichment of intestinal functions and enhanced maturation of hiPSC-derived intestinal epithelial cells grown in intestine-on-chip relative to organoids and Transwells

Lastly, we wanted to investigate the biological processes regulated by the culture environment of conventionally used intestinal model systems. We recently demonstrated that exposure to the EM-DM + D + P condition in a microfluidic hiPSC-derived intestine-on-chip system induces an epithelial composition that resembles the human small intestine (Moerkens et al., 2024). Here, we compared the transcriptomic profiles of hiPSC-derived intestinal epithelial cells grown as organoids (exposed to DM + D + P), in Transwell systems and in a commercially available intestine-on-chip system (both exposed to EM-DM + D + P) (Figures 5A and S2A). Pairwise analysis between the model systems and subsequent clustering of DEGs resulted in three clusters that reflect different expression profiles between the systems (Figure 5B; Data S1). The DEGs upregulated in intestinal organoids (cluster 1) encompass multiple genes related to mineral and metal metabolism, primarily metallothionein genes (e.g., *MT1F*, *MT1X*, and *MT1H*) (Thirumoorthy et al., 2007) (Figures 5B–5D). The DEGs upregulated in Transwell systems (cluster 3) are related to cell division, suggesting that, even though the Transwell and intestine-on-chip systems were both exposed to the EM-DM + D + P condition, the microenvironment of the intestine-on-chip might accelerate epithelial differentiation (Figures 5B–5D). In addition, processes related to extracellular matrix organization (including genes *TGFB1*, *TGFB2*, *COL2A1*, *COL4A6*, *LAMB1*, *LAMB2*, and *ACTA2*) and neurogenesis (e.g., *TUBB2B*, *NCAM1*, and *RGMA*) are enriched in Transwell systems (Elmentaite et al., 2021; Fawkner-Corbett et al., 2021; Holloway et al., 2021) (Figures 5B–5D). Accordingly, we observed more VIM-expressing epithelial cells in Transwell systems compared to organoids and intestine-on-chip systems, suggesting a potential epithelial-to-mesenchymal transition, as observed in a previous study (Moerkens et al., 2024) (Figure S2B). Genes specifically upregulated in intestine-on-chip systems (cluster 2) primarily included enterocyte-associated genes related to small intestinal functions such as nutrient metabolism (e.g., *ANPEP*, *SI*, *LCT*, *MGAM*, *PEPD*, *ALPI*, *LIPA*, and *TMPRSS15*), nutrient transport (e.g., *SLC2A2*, *SLC2A5*, *SLC15A1*, *SLC26A3*, *APOA1*, *APOB*, and *FABP2*), and xenobiotic metabolism (e.g., *CYP3A4*, *CYP3A5*, *CYP3A7*,

Figure 5. Enhanced intestinal functions and maturation in intestine-on-chips relative to organoids and Transwell systems
(A) Schematic of the experimental setup. Representative bright-field and immunofluorescent confocal image of cross-sectional slices of the intestine-on-chip system exposed to the EM-DM + D + P condition, stained for CDX2 (intestinal epithelium) and RBP2 (enterocyte). hiPSC, human induced pluripotent stem cell; EM, expansion medium; DM + D + P, differentiation medium + DAPT + PD0325901.
(B) Heatmap of average expression of the DEGs between organoids (DM + D + P), Transwell, and intestine-on-chip systems (both EM-DM + D + P), clustered by expression profile. Color represents row *Z* score. Columns correspond to donors (left = 1, middle = 2, and right = 3).
(C) Network of enriched biological processes, including top 20 with lowest adjusted *p* value per cluster. Node size reflects the number of DEGs per biological process, and edge thickness indicates the number of shared DEGs. ECM, extracellular matrix.
(D) Heatmap of average expression of DEGs selected from enriched processes described in (C). Color represents column *Z* score. Rows correspond to donors (top = 1, middle = 2, and bottom = 3).
(E) Scaled distance between epithelial cells of the human small intestine (columns; data from Gut Cell Atlas [Elmentaite et al., 2021, 2020] and Ramírez-Sánchez et al. [Ramírez-Sánchez et al., 2024] as dataset 2) and hiPSC-derived intestinal epithelial cells (rows). Distances were generated based on intestinal epithelial marker genes derived using the Gut Cell Atlas data. Smaller distance between samples (yellow values) indicates higher similarity, and larger distance (blue values) indicates lower similarity. Color represents column *Z* score. Rows correspond to donors as in (D), and columns correspond to donors present in the reference datasets.

CYP1A1, *CYP2C9*, *CYP2C18*, *CYP2D6*, *CYP2J2*, *UGT1A1*, *MAOB*, *ABCC2*, and *ABCB1*) (Burclaff et al., 2022; Fritz et al., 2019; Murata et al., 2023; Wang et al., 2020) (Figures 5B–5D). Strikingly, DEGs in clusters 1 (enhanced in organoids) and 3 (enhanced in Transwell systems) were enriched for processes related to embryonic development of the epithelium and differentiation of non-intestinal tissues (Figures 5B–5D). The expression of homeobox genes (e.g., *HOXA9*, *HOXB2*, *HOXB4*, and *HOXA1*) and genes involved in hedgehog signaling (e.g., *SHH*, *IHH*, *SMO*, *HES1*, and *SUFU*), instrumental for patterning differentiation of tissues in mammalian embryos (Ingham and McMahon, 2001; Mark et al., 1997), contributed to the identification of these processes (Figure 5D). This suggests that the epithelial tissue of organoids and in Transwell systems display a more fetal phenotype and resemble an earlier state of intestinal development compared to the tissue in intestine-on-chip systems.

To test whether the intestine-on-chip promotes intestinal epithelial maturation, we compared the transcriptome data of the organoids, Transwell, and intestine-on-chip systems to publicly available transcriptome data from human small intestinal epithelial cells at different developmental stages from the Gut Cell Atlas (Elmentaite et al., 2020, 2021, 2020; Ramírez-Sánchez et al., 2024). Indeed, organoids displayed the smallest distance and thus the highest resemblance to the fetal small intestine when compared to Transwell and intestine-on-chip systems, as determined by a distance analysis based on genes characteristic of human small intestinal epithelial cells (Figure 5E). Transwell systems did not show a specific resemblance to any intestinal developmental stage relative to the other two systems, while the intestine-on-chip systems showed the highest similarity to pediatric and adult human small intestinal expression profiles (Figure 5E).

According to these data, the intestine-on-chip drives the gene expression profile of hiPSC-derived intestinal epithelial cells toward processes related to nutrient and xenobiotic metabolism and more mature stages of small intestinal development (pediatric and adult), whereas organoids and Transwell systems may provide a better representation of fetal intestinal processes, including embryonic epithelial patterning, and specific processes such as mineral metabolism (organoids) and non-epithelial processes such as neurogenesis (Transwell systems).

DISCUSSION

Many studies have investigated the effect of *in vitro* culture conditions on ASC-derived intestinal epithelial cells, but only limited data are available for hiPSC-derived cells (Beumer and Clevers, 2021; Kasendra et al., 2020; Mitrofanova et al., 2024; Pleguezuelos-Manzano et al., 2020; Wang et al., 2017; Workman et al., 2018). In this study, we investigated the controlled induction of hiPSC-derived intestinal epithelial cell types and the biological processes induced by the culture microenvironment of organoids, Transwell, and intestine-on-chip systems. We found that hiPSC-derived intestinal epithelial cells can be steered toward specific lineages using modulators of the WNT, BMP, NOTCH, and MAPK pathways, previously described to control ASC-derived intestinal epithelial cell type specification (Beumer and Clevers, 2021; Pleguezuelos-Manzano et al., 2020). These principles can be applied to steer hiPSC-derived intestinal tissues toward a desired epithelial composition, regardless of the model system used. The levels of MUC2-positive goblet cells induced by 5-day exposure to the DM and DM + D conditions (20%–25%) resemble the abundance of goblet cells in the human large intestine (~20%), while the DM + D + P condition results in levels between 5% and 10%, closer to that of the small intestine (~5%) (Nahon et al., 2024; Wang et al., 2020). The levels of CHGA-positive enteroendocrine cells induced in the DM + D + P condition (below 1%) are lower than those previously reported by using MAPK inhibition in human ASC-derived intestinal organoids (10%–20%) (Beumer et al., 2018; Clevers and Beumer, 2020) but fall in the range reported for the human intestine (0%–1%) (Nahon et al., 2024; Wang et al., 2020). To balance epithelial proliferation and differentiation, and thus increase epithelial diversity and physiological relevance, basolateral exposure to the EM condition and apical exposure to a type of DM can be applied in both Transwell and intestine-on-chip systems (Moerkens et al., 2024). The fact that this works in the static and dynamic environment of both systems demonstrates that continuous renewal of medium in a microfluidic system is not required.

When differentiating hiPSCs to intestinal cells, mesenchymal cells co-develop and expand in EM conditions (Moerkens et al., 2024; Spence et al., 2011). In the current study, we aimed to investigate hiPSC-derived intestinal epithelial cells specifically and set out to generate intestinal tissues with negligible numbers of mesenchymal cells through selection of EPCAM-positive cells and consecutive rounds of single-cell passaging. Nevertheless, we still identified some cells expressing the mesenchymal marker VIM and observed that VIM expression levels correlated positively with the expression of cell cycle, stem cell, and Paneth cell genes and negatively with epithelial differentiation in the different donors in the Transwell system. Moreover, the intestine-on-chip exposed to EM-DM + D + P in this study had a lower expression of genes associated with the cell cycle and Paneth cells and a lower tissue height when compared to an intestine-on-chip in the same medium condition that included mesenchymal and neural cells

that we described in a previous study (Moerkens et al., 2024). These data suggest that hiPSC-derived epithelial cells may depend on mesenchymal-derived factors to sustain a proliferative and potentially stem cell- and Paneth-like state. Inclusion of mesenchymal cells or addition of mesenchymal-derived factors (such as WNT ligands or R-spondins) to the EM condition may help facilitate the long-term maintenance of dividing hiPSC-derived intestinal epithelial cells.

As shown here, the unique culture environments of model systems change the cellular and gene expression profiles of epithelial cells, likely due to differences such as the physiological shear stress induced by continuous fluid flow, stiffness profiles induced by embedding in extracellular matrix, or the configuration into multiple compartments, which enables dual exposure to EM and DM. The cells in the Transwell system showed an increased expression of neuron-associated and extracellular matrix genes and had a higher expression of epithelial cells expressing VIM. This might indicate that the Transwell microenvironment or the longer culturing times required to establish a polarized epithelial barrier in this static environment enhance the presence of mesenchymal and neural cells and phenotypes resembling epithelial-to-mesenchymal transition, as we observed earlier in intestine-on-chip systems (Moerkens et al., 2024). Intestinal organoids displayed transcriptional profiles indicating an upregulation of mineral absorption and metallothioneins, which protect against metal toxicity in the human intestine (Thirumoorthy et al., 2007). Potentially, this results from an enhanced induction of metallothionein-expressing enterocytes, which co-develop along with more classical enterocytes in hiPSC-derived intestinal systems (Moerkens et al., 2024).

Although we could induce expression profiles reflecting well-established intestinal metabolic processes in organoids and Transwell systems upon exposure to (EM-)DM + D + P, they were further enhanced in the dynamic environment of the intestine-on-chip. These included genes involved in the digestion and transport of carbohydrates, proteins, lipids, and ions (Burclaff et al., 2022; Wang et al., 2020) and the conversion of therapeutic compounds in the human intestine, specifically cytochrome P450 enzymes from the CYP3A and CYP2C families (Thelen and Dressman, 2009). Also genes known to display high inter-individual genetic variability (e.g., lactase, *LCT*, and *CYP2D6*) were expressed (Ingelman-Sundberg, 2005; Walther et al., 2019), emphasizing the value of hiPSC-derived intestinal epithelial cells for personalized modeling of intestinal digestion and drug metabolism under relevant culture conditions. The elevated expression of nutrient and drug metabolism upon exposure to continuous fluid flow was in concordance with findings from studies using human ASC-derived intestinal epithelial cells: genes associated with lipid, protein, and xenobiotic metabolism were upregulated in a human duodenum-derived intestine-on-chip when compared to organoids (Kasendra et al., 2020) and in monolayers of duodenal epithelial cells after exposure to rotational flow profiles (Sugimoto et al., 2021). Moreover, an ASC-derived colon intestine-on-chip showed upregulation of CYP3A4 when compared to organoids (Mitrofanova et al., 2024). In addition to enhanced enterocyte functions, microfluidic intestine-on-chip systems show potential to study nutrient and drug metabolism and absorption given their easy access to the basolateral and apical side of the epithelial barrier, enlarged epithelial surface area due to the presence of villus-like folds, physiological barrier integrity (Moerkens et al., 2024), and ability to sustain dividing and differentiated epithelial cell types. Moreover, they confer a unique opportunity to study the bioavailability of compounds after oral intake and first-pass metabolism when coupled to a liver-on-chip containing the same genetic background.

The relative fetal state of hiPSC-derived intestinal organoids was previously improved by a 6- to 12-week transplantation in mice (Childs et al., 2023; Finkbeiner et al., 2015; Watson et al., 2014). Our study shows that maturation and specific intestinal processes can also be induced by exposure to DM-type conditions. Importantly, we demonstrate that the more physiological environment in the intestine-on-chip can improve the overall maturation state of hiPSC-derived intestinal epithelial cells *in vitro*. Relative to the other model systems, the gene expression profiles in the intestine-on-chip showed more similarity to later developmental stages of the human small intestine (pediatric and adult), whereas the profiles of organoids were most similar to fetal stages. Indeed, genes involved in embryonic developmental processes, particularly homeobox genes and genes involved in hedgehog signaling, were downregulated in intestine-on-chip systems when compared to organoids and Transwell systems. The enhanced maturation in the intestine-on-chip system was also achieved with a shorter culturing time of 11 days, as compared to 19 days in Transwell systems and 12 days as organoids.

In conclusion, exposure to activators and inhibitors of the WNT, BMP, NOTCH, and MAPK pathways allows the controlled induction of hiPSC-derived intestinal epithelial lineages. While hiPSC-derived intestinal epithelial cells grown as organoids or in Transwell systems might represent fetal or developmental intestinal processes, intestine-on-chip systems could be relevant for modeling more mature intestinal phenotypes and digestive processes of the human small intestine in a personalized manner.

METHODS

Cell lines and culturing conditions

hiPSC lines were generated from urine-derived renal epithelial cells of four donors (two male and two female) by the induced pluripotent stem cell (iPSC)/CRISPR facility of the European Research Institute for the Biology of Aging (ERIBA) and University medical Center Groningen (UMCG) using a lentiviral vector described earlier (Warlich et al., 2011). The hiPSC lines were maintained in mTeSR Plus (STEMCELL Technologies, #05825) on plates coated with human embryonic stem cell-qualified Matrigel (Corning #354277) in a humidified environment at 37°C in 5% CO_2 and passaged using ReLeSR (STEMCELL Technologies, #05872). The experiments with hiPSC lines were approved by the ethics committee of the University Medical Center Groningen (document no. METC 2013/440), and written consent was obtained from the donors.

Generation of intestinal epithelial cells from hiPSCs

The procedures to differentiate intestinal organoids from hiPSCs, to select epithelial cells from intestinal organoids, and to cryopreserve intestinal epithelial cells were described previously (Moerkens et al., 2024). Intestinal epithelial cells were then thawed, resuspended in basement membrane Matrigel (Corning #354234), and plated in domes in a 24-well plate (Thermo Fisher Scientific#142475) at a concentration of 1,000 cells/μL Matrigel. After 10 min of incubation at 37°C, Matrigel domes had solidified and were overlaid with Advanced DMEM/F12 (Thermo Fisher Scientific #12634010) supplemented with L-glutamine (2 mM, Thermo Fisher Scientific #25030081), penicillin-streptomycin (100 units/mL; 100 μg/mL respectively, Thermo Fisher Scientific # 15140122), Noggin (100 ng/mL, R&D Systems, #6057-NG/CF), epidermal growth factor (EGF) (100 ng/mL, R&D Systems, #236-EG), CHIR99021 (2 μM, Tocris, #4423), B27 (1x, Thermo Fisher Scientific #17504044), SB202190 (10 μM, Tocris #1264/10), A83-01 (500 nM, Tocris #2939/10), hereafter named "expansion medium" (EM), and Y-27632 (10 μM, Tocris #1254/10). After 48 h, the medium was replaced with fresh EM to remove Y-27632, and EM was replaced every other day. For 4–5 weeks, human intestinal organoids were grown and passaged weekly as fragments or single cells to further remove mesenchymal cells that co-develop during differentiation and expand the intestinal epithelial cells. For fragment passaging, organoids were released from Matrigel domes by mechanical dislodgement of domes and repeated pipetting in cold Advanced DMEM/F12. After centrifugation ($400 \times g$, 5 min, 4°C), the Matrigel was removed and organoids were fragmented by repeated pipetting. Fragments were resuspended in fresh Matrigel and handled as previously described. For single-cell passaging, the same procedure was followed, but fragments were dissociated to a single-cell suspension using TrypLE Select (Thermo Fisher Scientific #12563029) and then passed through a 70-μm filter before seeding in Matrigel domes.

Maintaining intestinal epithelial cells as organoids in Transwells and in intestine-on-chips

To seed organoids, intestinal epithelial cells were resuspended as single cells in basement membrane Matrigel at a concentration of 500 cells/μL and plated in domes overlaid with EM containing Y-27632 as described before. For immunofluorescent microscopy, domes were plated in Nunc Lab-Tek chamber slide systems (Thermo Fisher Scientific #178599PK) and spread over the surface of the well before solidification. To seed Transwell systems (Corning #3401), inserts were coated with basement membrane Matrigel (83 μg/mL) diluted in Advanced DMEM/F12 and incubated (1 h, RT). After aspirating the coating, EM with Y-27632 was added to the bottom reservoir, and intestinal epithelial cells in EM with Y-27632 were seeded onto inserts (2×10^5 cells/insert). Intestine-on-chips (Polydimethylsiloxaan [PDMS]-based Chip-S1, Emulate Inc Boston, MA) containing two parallel microfluidic channels (top channel: 1,000 × 1,000 μm, bottom channel: 1,000 × 200 μm, channel length: 28 mm) were seeded as described previously (Moerkens et al., 2024). In short, the two microfluidic channels were activated, coated with basement membrane Matrigel (83 μg/mL) diluted in Advanced DMEM/F12, and 3×10^5 intestinal epithelial cells suspended in EM containing Y-27632 were seeded in the top channel. The chips were incubated for 3 h until cells were attached to the membrane, washed gently, and connected to the Emulate instrument. The flow rate of the media within both channels was 30 μL/hour, which imposes a wall shear stress on the cells of 0.05 mPa in the top channel and 1.0 mPa in the bottom channel. Culture medium added to the chips was always first equilibrated. Cells were grown in EM for 6 (intestine-on-chip), 7 (organoids), or 14 days (Transwell), determined by stabilized barrier integrity (intestine-on-chip [Moerkens et al., 2024] and Transwell) or sufficient spheroid size (Clevers and Beumer, 2020), and thereafter exposed to different medium conditions for 5 days: EM, DM, DM + D, or DM + D + P. DM was composed of Advanced DMEM/F12 supplemented with L-glutamine (2 mM), penicillin-streptomycin (100 units/mL and 100 μg/mL, respectively), EGF (100 ng/mL), and B27 (1x). DM + D was composed of DM including DAPT (10 μM, Tocris #2634). DM + D + P was composed of DM + D including PD0325901 (0.1 μM, Tocris #4192). The concentration of dissolvent was equal in all medium conditions. All systems were maintained in a humidified environment at 37°C in 5% CO_2, and respective media were refreshed every

2 days for organoids and Transwell systems and continuously for the intestine-on-chip systems.

Immunofluorescent microscopy and flow cytometry

Cells were fixed and stained for immunofluorescent and flow cytometry analysis as detailed in supplemental methods.

Differential gene expression and pathway enrichment analysis

RNA was harvested from organoids, Transwells, and intestine-on-chips and sequenced, and gene expression was quantified as described in supplemental methods. The DEGs between different conditions were identified using the R package DESeq2 (version 1.44.0) including donor as covariate in the differential expression (DE) model. Genes with 10 or more reads in at least 3 samples were filtered. Multiple pairwise comparisons were performed to analyze differences between medium conditions or model systems in the relevant subsets of the data, as described in Data S1. DEGs were filtered on having an absolute log2FoldChange $\geq$ 1 and an adjusted p value < 0.01 and presented in heatmaps as normalized or scaled counts. Normalized and scaled expression levels of DEGs were k-means clustered using the hclust() and dist() functions from R. Biological processes enriched in the resulting clusters were identified using the R package clusterProfiler (version 4.12.0) using the Gene Ontology: Biological Processes database. p values were adjusted using the Benjamini-Hochberg procedure. Data were visualized using clusterProfiler's emapplot function, showing the top 20 biological processes per cluster based on adjusted p value. Distance matrix analysis was performed as detailed in supplemental methods.

Quantification and statistical analysis

The data were presented as median with interquartile range or mean with standard deviation, as indicated in figure captions. Significant differences between the media conditions were determined using a one-way analysis of variance test with the Tukey multiple comparisons test. Differences between groups were considered statistically significant when p value < 0.05. Rstudio with the R package rstatix was used for statistical analysis (Kassambara, 2023).

RESOURCE AVAILABILITY

Lead contact

Further information and requests should be directed to the corresponding author, Sebo Withoff (s.withoff@umcg.nl).

Materials availability

This study did not generate new unique reagents.

Data and code availability

- The RNA sequencing data generated in this study are available at the European Genome-Phenome Archive (EGA): EGAD50000001918.
- The code required to reproduce the RNA sequencing analysis can be found at https://github.com/umcg-immunogenetics/Organoid_Transwell_Chip_Moerkens_2024.

ACKNOWLEDGMENTS

This work was supported by the Netherlands Organ-on-Chip Initiative, an NWO Gravitation project (024.003.001) funded by the Ministry of Education, Culture, and Science of the government of the Netherlands (R. Moerkens, C.W., and S.W.). J.M. is supported by a PhD scholarship from the Graduate School of Medical Sciences, University of Groningen. I.H.J. is supported by a Rosalind Franklin Fellowship from the University of Groningen and an NWO VIDI grant (016.171.047). We thank Kate Mc Intyre for editing the manuscript. We thank the iPSC/CRISPR facility, UMCG Microscopy and Imaging Center, and Flow Cytometry Unit of the University Medical Center Groningen for their support and services. We thank Emulate Inc (Boston, USA) for kindly providing the Human Emulation System. We thank the members of the Netherlands Organ-on-Chip Initiative for insightful discussions and support regarding the implementation of organ-on-chip technology.

AUTHOR CONTRIBUTIONS

R. Moerkens and J.M. designed and executed experiments and analyzed experimental data. R. Moerkens wrote the manuscript. M.B. analyzed RNA sequencing data. E.S. and R. Modderman assisted in executing experiments. R. Moerkens, J.M., A.D.R.-S., J.P., C.P.-M., and R.J.B. (co-)developed and/or were consulted for methodologies. C.W. assisted in conceiving the project. I.H.J. and S.W. supervised the project and provided feedback on experiment design, data analysis, and writing.

DECLARATION OF INTERESTS

The authors declare no competing interests.

SUPPLEMENTAL INFORMATION

Supplemental information can be found online at https://doi.org/10.1016/j.stemcr.2025.102715.

Received: October 11, 2024
Revised: October 18, 2025
Accepted: October 18, 2025
Published: November 13, 2025

REFERENCES

Afroze, S., Meng, F., Jensen, K., McDaniel, K., Rahal, K., Onori, P., Gaudio, E., Alpini, G., Glaser, S.S., and Glaser, S.S. (2013). The physiological roles of secretin and its receptor. Ann. Transl. Med. *1*, 29. https://doi.org/10.3978/j.issn.2305-5839.2012.12.01.

Beumer, J., Artegiani, B., Post, Y., Reimann, F., Gribble, F., Nguyen, T.N., Zeng, H., Van den Born, M., Van Es, J.H., Clevers, H., and Clevers, H. (2018). Enteroendocrine cells switch hormone expression along the crypt-to-villus BMP signaling gradient. Nat. Cell Biol. *20*, 909–916. https://doi.org/10.1038/s41556-018-0143-y.

Beumer, J., and Clevers, H. (2021). Cell fate specification and differentiation in the adult mammalian intestine. Nat. Rev. Mol. Cell Biol. *22*, 39–53. https://doi.org/10.1038/s41580-020-0278-0.

Burclaff, J., Bliton, R.J., Breau, K.A., Ok, M.T., Gomez-Martinez, I., Ranek, J.S., Bhatt, A.P., Purvis, J.E., Woosley, J.T., Magness, S.T., and Magness, S.T. (2022). A Proximal-to-Distal Survey of Healthy Adult Human Small Intestine and Colon Epithelium by Single-Cell Transcriptomics. Cmgh *13*, 1554–1589. https://doi.org/10.1016/j.jcmgh.2022.02.007.

Childs, C.J., Poling, H.M., Chen, K., Tsai, Y., Wu, A., Vallie, A., Eiken, M.K., Huang, S., Sweet, C.W.,, Schreiner, R., et al. (2023). Coordinated differentiation of human intestinal organoids with functional enteric neurons and vasculature. Cell Stem Cell *32*, 640–651.e9. https://doi.org/10.1101/2023.11.06.565830.

Clevers, H., and Beumer, J. (2020). Induction of different human enteroendocrine cells in intestinal organoids. Protocol Exchange, 1–9. https://dx.doi.org/10.21203/rs.3.nprot-6891/v1.

Eissa, N., Hussein, H., Hendy, G.N., Bernstein, C.N., and Ghia, J.-E. (2018). Chromogranin-A and its derived peptides and their pharmacological effects during intestinal inflammation. Biochem. Pharmacol. *152*, 315–326.

Elmentaite, R., Kumasaka, N., Roberts, K., Fleming, A., Dann, E., King, H.W., Kleshchevnikov, V., Dabrowska, M., Pritchard, S., Bolt, L., et al. (2021). Cells of the human intestinal tract mapped across space and time. Nature *597*, 250–255. https://doi.org/10.1038/s41586-021-03852-1.

Elmentaite, R., Ross, A.D.B., Roberts, K., James, K.R., Ortmann, D., Gomes, T., Nayak, K., Tuck, L., Pritchard, S., Bayraktar, O.A., et al. (2020). Single-Cell Sequencing of Developing Human Gut Reveals Transcriptional Links to Childhood Crohn's Disease. Dev. Cell *55*, 771–783.e5. https://doi.org/10.1016/j.devcel.2020.11.010.

Fawkner-Corbett, D., Antanaviciute, A., Parikh, K., Jagielowicz, M., Gerós, A.S., Gupta, T., Ashley, N., Khamis, D., Fowler, D., Morrissey, E., et al. (2021). Spatiotemporal analysis of human intestinal development at single-cell resolution. Cell *184*, 810–826.e23. https://doi.org/10.1016/j.cell.2020.12.016.

Finkbeiner, S.R., Hill, D.R., Altheim, C.H., Dedhia, P.H., Taylor, M.J., Tsai, Y.H., Chin, A.M., Mahe, M.M., Watson, C.L., Freeman, J.J., et al. (2015). Transcriptome-wide Analysis Reveals Hallmarks of Human Intestine Development and Maturation In Vitro and In Vivo. Stem Cell Rep. *4*, 1140–1155. https://doi.org/10.1016/j.stemcr.2015.04.010.

Fritz, A., Busch, D., Lapczuk, J., Ostrowski, M., Drozdzik, M., and Oswald, S. (2019). Expression of clinically relevant drug-metabolizing enzymes along the human intestine and their correlation to drug transporters and nuclear receptors: An intra-subject analysis. Basic Clin. Pharmacol. Toxicol. *124*, 245–255.

Guengerich, F.P. (1999). Cytochrome P-450 3A4: regulation and role in drug metabolism. Annu. Rev. Pharmacol. Toxicol. *39*, 1–17.

Holloway, E.M., Czerwinski, M., Tsai, Y.H., Wu, J.H., Wu, A., Childs, C.J., Walton, K.D., Sweet, C.W., Yu, Q., Glass, I., et al. (2021). Mapping Development of the Human Intestinal Niche at Single-Cell Resolution. Cell Stem Cell *28*, 568–580.e4. https://doi.org/10.1016/j.stem.2020.11.008.

Ingelman-Sundberg, M. (2005). Genetic polymorphisms of cytochrome P450 2D6 (CYP2D6): clinical consequences, evolutionary aspects and functional diversity. Pharmacogenomics J. *5*, 6–13.

Ingham, P.W., and McMahon, A.P. (2001). Hedgehog signaling in animal development: Paradigms and principles. Genes Dev. *15*, 3059–3087. https://doi.org/10.1101/gad.938601.

Jang, C., Hui, S., Lu, W., Cowan, A.J., Morscher, R.J., Lee, G., Liu, W., Tesz, G.J., Birnbaum, M.J., Rabinowitz, J.D., and Rabinowitz, J.D. (2018). The Small Intestine Converts Dietary Fructose into Glucose and Organic Acids. Cell Metab. *27*, 351–361.e3. https://doi.org/10.1016/j.cmet.2017.12.016.

Kasendra, M., Luc, R., Yin, J., Manatakis, D.V., Kulkarni, G., Lucchesi, C., Sliz, J., Apostolou, A., Sunuwar, L., Obrigewitch, J., et al. (2020). Duodenum intestine-chip for preclinical drug assessment in a human relevant model. eLife *9*, e50135-23. https://doi.org/10.7554/eLife.50135.

Kassambara, A. (2023). rstatix: Pipe-Friendly Framework for Basic Statistical Tests (R package). https://rpkgs.datanovia.com/rstatix/.

Mark, M., Rijli, F.M., and Chambon, P. (1997). Homeobox Genes in Embryogenesis and Pathogenesis. Pediatr. Res. *42*, 421–429.

Mitrofanova, O., Broguiere, N., Nikolaev, M., and Lutolf, M.P. (2024). Bioengineered human colon organoids with in vivo - like complexity and function. Cell Stem Cell *31*, 1175–1186.e7. https://doi.org/10.1016/j.stem.2024.05.007.

Moerkens, R., Mooiweer, J., Ramírez-Sánchez, A.D., Oelen, R., Franke, L., Wijmenga, C., Barrett, R.J., Jonkers, I.H., Withoff, S., and Withoff, S. (2024). An iPSC-derived small intestine-on-chip with self-organizing epithelial, mesenchymal and neural cells. Cell Rep. *43*, 114247. https://doi.org/10.1016/j.celrep.2024.114247.

Muntjewerff, E.M., Tang, K., Lutter, L., Christoffersson, G., Nicolasen, M.J.T., Gao, H., Katkar, G.D., Das, S., Ter Beest, M., Ying, W., et al. (2021). Chromogranin A regulates gut permeability via the antagonistic actions of its proteolytic peptides. Acta Physiol. *232*, e13655. https://doi.org/10.1111/apha.13655.Chromogranin.

Murata, M., Fujioka, H., Yokota, J., Okada, K., Yamashita, T., Hirayama, D., Kawakami, K., Morinaga, G., Saito, A., Nakase, H., et al. (2023). Regional Transcriptomics and Proteomics of Pharmacokinetics-Related Genes in Human Intestine. Mol. Pharm. *20*, 2876–2890.

Nahon, D.M., Moerkens, R., Aydogmus, H., Lendemeijer, B., Martínez-Silgado, A., Stein, J.M., Dostanić, M., Frimat, J.P., Gontan, C., de Graaf, M.N.S., et al. (2024). Standardizing designed and emergent quantitative features in microphysiological systems. Nat. Biomed. Eng. *8*, 941–962. https://doi.org/10.1038/s41551-024-01236-0.

Ohno, T., Mochiki, E., and Kuwano, H. (2010). The roles of motilin and ghrelin in gastrointestinal motility. Int. J. Pept. *2010*, 820794. https://doi.org/10.1155/2010/820794.

Pleguezuelos-Manzano, C., Puschhof, J., van den Brink, S., Geurts, V., Beumer, J., and Clevers, H. (2020). Establishment and Culture of

Human Intestinal Organoids Derived from Adult Stem Cells. Curr. Protoc. Immunol. *130*, e106–e124. https://doi.org/10.1002/cpim.106.

Raab, S., Klingenstein, M., Liebau, S., and Linta, L. (2014). A Comparative View on Human Somatic Cell Sources for iPSC Generation. Stem Cells Int. *2014*, 768391. https://doi.org/10.1155/2014/768391.

Ramírez-Sánchez, A.D., Zühlke, S., Aguirre-Gamboa, R., Vochteloo, M., Franke, L., Lundin, K.E.A., Withoff, S., and Jonkers, I.H. (2024). Gene expression and eQTL analysis reflect the heterogeneity in the inflammatory status of the duodenal epithelial lining in coeliac disease. Preprint at bioRxiv. https://doi.org/10.1101/2024.02.29.582756.

Sato, T., Stange, D.E., Ferrante, M., Vries, R.G.J., van Es, J.H., van den Brink, S., Van Houdt, W.J., Pronk, A., Van Gorp, J., Siersema, P.D., et al. (2011). Long-term Expansion of Epithelial Organoids From Human Colon, Adenoma, Adenocarcinoma, and Barrett's Epithelium. Gastroenterology *141*, 1762–1772. https://doi.org/10.1053/J.GASTRO.2011.07.050.

Spence, J.R., Mayhew, C.N., Rankin, S.A., Kuhar, M.F., Vallance, J.E., Tolle, K., Hoskins, E.E., Kalinichenko, V.V., Wells, S.I., Zorn, A.M., et al. (2011). Directed differentiation of human pluripotent stem cells into intestinal tissue in vitro. Nature *470*, 105–109. https://doi.org/10.1038/nature09691.

Sugimoto, S., Kobayashi, E., Fujii, M., Ohta, Y., Arai, K., Matano, M., Ishikawa, K., Miyamoto, K., Toshimitsu, K., Takahashi, S., et al. (2021). An organoid-based organ-repurposing approach to treat short bowel syndrome. Nature *592*, 99–104. https://doi.org/10.1038/s41586-021-03247-2.

Takahashi, K., Tanabe, K., Ohnuki, M., Narita, M., Ichisaka, T., Tomoda, K., and Yamanaka, S. (2007). Induction of Pluripotent Stem Cells from Adult Human Fibroblasts by Defined Factors. Cell *131*, 861–872. https://doi.org/10.1016/J.CELL.2007.11.019.

Thelen, K., and Dressman, J.B. (2009). Cytochrome P450-mediated metabolism in the human gut wall. J. Pharm. Pharmacol. *61*, 541–558.

Thirumoorthy, N., Manisenthil Kumar, K.T., Shyam Sundar, A., Panayappan, L., and Chatterjee, M. (2007). Metallothionein : An overview. World J. Gastroenterol. *13*, 993–996.

Tracy, T.S., Chaudhry, A.S., Prasad, B., Thummel, K.E., Schuetz, E.G., Zhong, X.B., Tien, Y.C., Jeong, H., Pan, X., Shireman, L.M., et al. (2016). Interindividual Variability in Cytochrome P450–Mediated Drug Metabolism. Drug Metab. Dispos. *44*, 343–351.

Walther, B., Lett, A.M., Bordoni, A., Tomás-Cobos, L., Nieto, J.A., Dupont, D., Danesi, F., Shahar, D.R., Echaniz, A., Re, R., et al. (2019). GutSelf: Interindividual Variability in the Processing of Dietary Compounds by the Human Gastrointestinal Tract. Mol. Nutr. Food Res. *63*, e1900677. https://doi.org/10.1002/mnfr.201900677.

Wang, Y., Gunasekara, D.B., Reed, M.I., DiSalvo, M., Bultman, S.J., Sims, C.E., Magness, S.T., Allbritton, N.L., and Allbritton, N.L. (2017). A microengineered collagen scaffold for generating a polarized crypt-villus architecture of human small intestinal epithelium. Biomaterials *128*, 44–55. https://doi.org/10.1016/J.BIOMATERIALS.2017.03.005.

Wang, Y., Song, W., Wang, J., Wang, T., Xiong, X., Qi, Z., Fu, W., Yang, X., Chen, Y.G., and Chen, Y.G. (2020). Single-cell transcriptome analysis reveals differential nutrient absorption functions in the human intestine. J. Exp. Med. *217*, e20191130. https://doi.org/10.1084/jem_20191130.

Warlich, E., Kuehle, J., Cantz, T., Brugman, M.H., Maetzig, T., Galla, M., Filipczyk, A.A., Halle, S., Klump, H., Schöler, H.R., et al. (2011). Lentiviral vector design and imaging approaches to visualize the early stages of cellular reprogramming. Mol. Ther. *19*, 782–789. https://doi.org/10.1038/mt.2010.314.

Watson, C.L., Mahe, M.M., Múnera, J., Howell, J.C., Sundaram, N., Poling, H.M., Schweitzer, J.I., Vallance, J.E., Mayhew, C.N., Sun, Y., et al. (2014). An in vivo model of human small intestine using pluripotent stem cells. Nat. Med. *20*, 1310–1314. https://doi.org/10.1038/nm.3737.

Workman, M.J., Gleeson, J.P., Troisi, E.J., Estrada, H.Q., Kerns, S.J., Hinojosa, C.D., Hamilton, G.A., Targan, S.R., Svendsen, C.N., Barrett, R.J., and Barrett, R. (2018). Enhanced Utilization of Induced Pluripotent Stem Cell–Derived Human Intestinal Organoids Using Microengineered Chips. Cell. Mol. Gastroenterol. Hepatol. *5*, 669–677.e2.

Stem Cell Reports, Volume *20*

Supplemental Information

Gene expression profiling reveals enhanced nutrient and drug metabolism and maturation of hiPSC-derived intestine-on-chip relative to organoids and Transwells

Renée Moerkens, Joram Mooiweer, Eline Smits, Marijn Berg, Aarón D. Ramírez-Sánchez, Rutger Modderman, Jens Puschhof, Cayetano Pleguezuelos-Manzano, Robert J. Barrett, Cisca Wijmenga, Iris H. Jonkers, and Sebo Withoff

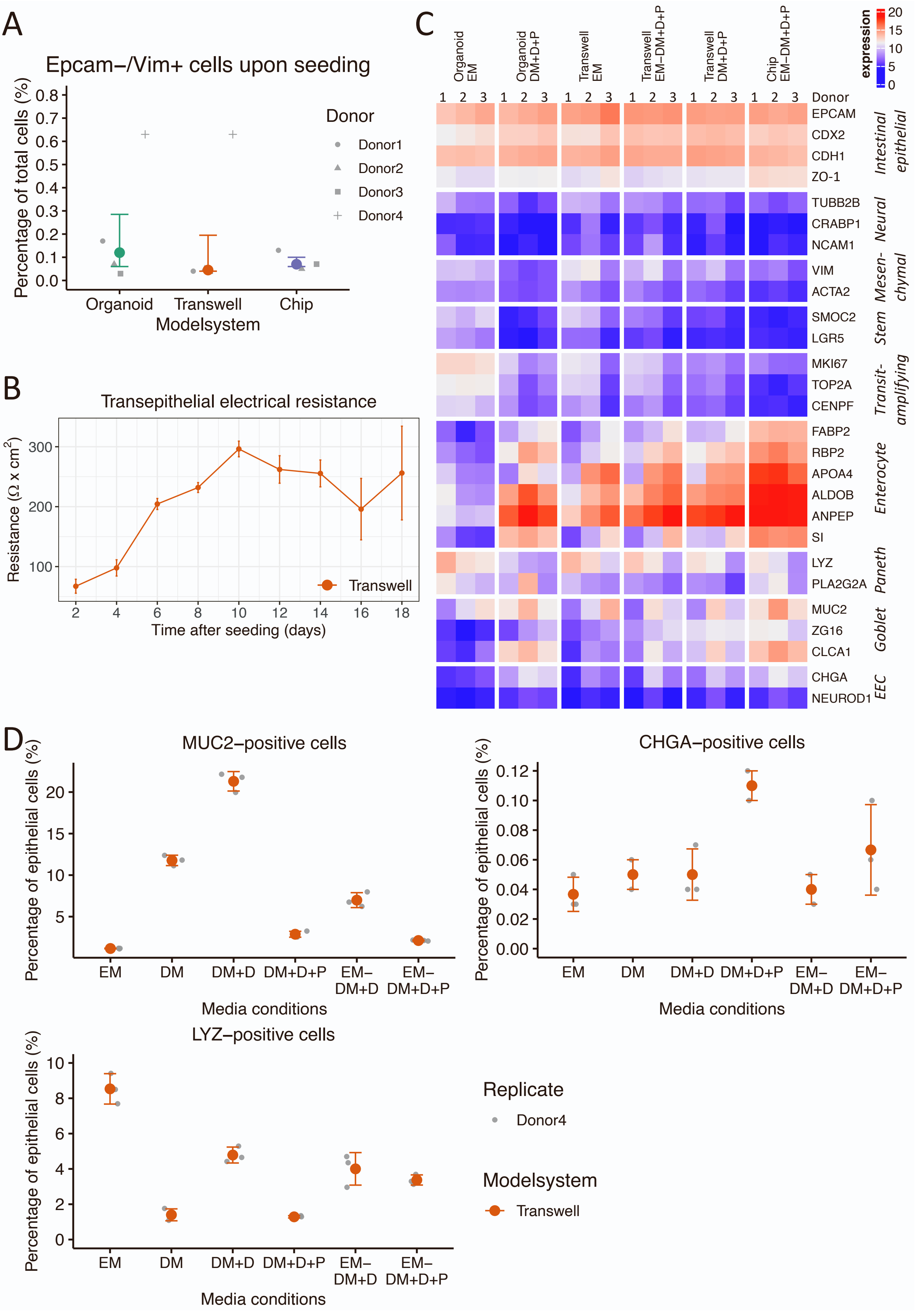
A
Epcam−/Vim+ cells upon seeding
Percentage of total cells (%)
0.8
0.7
0.6
0.5
0.4
0.3
0.2
0.1
0.0
Organoid
Transwell
Chip
Modelsystem
Donor
Donor1
Donor2
Donor3
Donor4
B
Transepithelial electrical resistance
Resistance (Ω x cm2)
300
200
100
Transwell
2
4
6
8
10
12
14
16
18
Time after seeding (days)
C
Organoid EM
Organoid DM+D+P
Transwell EM
Transwell EM−DM+D+P
Transwell DM+D+P
Chip EM−DM+D+P
expression
20
15
10
5
0
1 2 3
Donor
EPCAM
CDX2
CDH1
ZO-1
Intestinal epithelial
TUBB2B
CRABP1
NCAM1
Neural
VIM
ACTA2
Mesen-chymal
SMOC2
LGR5
Stem
MKI67
TOP2A
CENPF
Transit-amplifying
FABP2
RBP2
APOA4
ALDOB
ANPEP
SI
Enterocyte
LYZ
PLA2G2A
Paneth
MUC2
ZG16
CLCA1
Goblet
CHGA
NEUROD1
EEC
D
MUC2−positive cells
Percentage of epithelial cells (%)
20
15
10
5
EM
DM
DM+D
DM+D+P
EM−DM+D
EM−DM+D+P
Media conditions
CHGA−positive cells
0.12
0.10
0.08
0.06
0.04
0.02
0.00
LYZ−positive cells
10
8
6
4
2
0
Replicate
Donor4
Modelsystem
Transwell

Figure S1. Expression of epithelial-, mesenchymal- and neural-specific markers. (A) Flow cytometry quantification of Epcam-negative VIM-positive cells present upon seeding of the model systems, displayed as median with interquartile range of four donors. (B) Transepithelial electrical resistance (TEER) over time in Transwell systems, displayed as mean with standard deviation of three technical replicates. (C) Expression levels of genes associated with intestinal epithelial, mesenchymal and neural cells and multiple intestinal epithelial cell types in diverse model systems and medium conditions. EEC = enteroendocrine cell. Color scale represents normalized counts. (D) Flow cytometry quantification of the intra donor variation in cell type proportions, displayed as mean with standard deviation of four technical replicates.

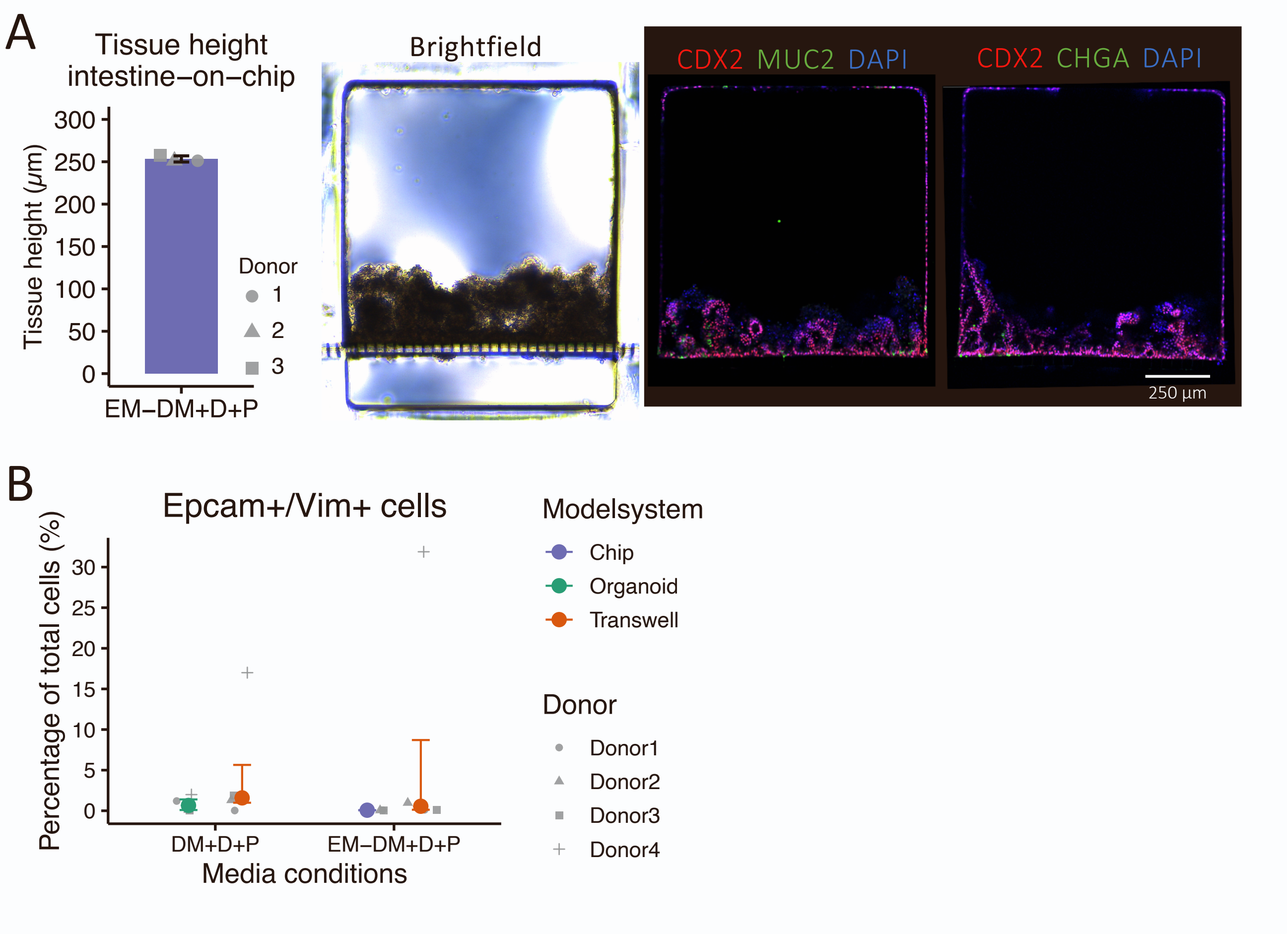

Figure S2. Characterization of the hiPSC-derived intestine-on-chip. (A) Tissue height quantification and representative brightfield and immunofluorescent confocal images of cross-sectional slices of intestine-on-chip systems exposed to the EM-DM+D+P condition, stained for CDX2 (intestinal epithelium), MUC2 (goblet cell) and CHGA (enteroendocrine cell). Tissue height quantification is displayed as mean with standard deviation of three donors. (B) Flow cytometry quantification of Epcam-positive VIM-positive cells in different model systems, displayed as median with interquartile range of four donors.

Table S1. Primary antibodies used for immunofluorescent microscopy

Antigen	Dilution	Host/Isotype	Catalogue number	Manufacturer
CHGA	1:100	Mouse IgG1	MA5-13096	ThermoFisher
MUC2	1:200	Mouse IgG1	ab118964	Abcam
LYZ	1:200	Mouse IgG2a	NB100-63062	Novus Biologicals
MKI67	1:200	Rabbit IgG	ab16667	Abcam
RBP2	1:100	Rabbit polyclonal	HPA035866	Sigma Aldrich
ZO-1	1:100	Rabbit IgG polyclonal	61-7300	ThermoFisher
CDX2	1:100	Goat IgG polyclonal	AF3665-SP	R&D systems

Table S2. Secondary antibodies used for immunofluorescent microscopy

Fluorophore	Dilution	Species reactivity	Catalogue number	Manufacturer
Alexa Fluor 488	1:250	Mouse IgG (H+L)	715-545-150	Jackson ImmunoResearch
Alexa Fluor 647	1:250	Goat IgG (H+L)	A-21447	ThermoFisher
Cy3	1:250	Rabbit IgG (H+L)	711-165-152	Jackson ImmunoResearch

Table S3. Antibodies used for flow cytometry

Antigen	Fluorophore	Dilution	Catalogue number	Manufacturer
VIM	Alexa Fluor 594	1:50	7675S	Cell Signaling Technology
EPCAM/CD326	BUV737	1:100	748397	BD Biosciences
CHGA	PE	1:1000	ab213341	Abcam
MUC2	PerCP	1:67	NBP2-34757PCP	Novus Biologicals
LYZ	Alexa Fluor 488	1:200	NB100-63062AF488	Novus Biologicals

Supplemental methods

Immunofluorescent microscopy

Cells were fixed in paraformaldehyde (PFA, 4% vol/vol DPBS, ThermoFisher #28908) for 10 min (Transwell and intestine-on-chip) or 60 min (organoids), washed and stored in DPBS at 4 °C. Cross-sectional slices of intestine-on-chip systems (200 µm thick) were generated using a vibratome (VT1000S, Leica) as described previously (Moerkens et al., 2024) and Transwell inserts were removed and divided in quarters using a scalpel and microtome blade. All samples were permeabilized in Triton X-100 (0.1% vol/vol DPBS, Sigma Aldrich #T8787) for 10-15 min (Transwell systems) or 30 min (organoids and intestine-on-chip systems) and blocked with bovine serum albumin (BSA, 3% weight/vol DPBS, Sigma Aldrich #A2153) for 1 hour. Primary antibodies diluted in BSA solution were applied overnight at 4 °C. Samples were washed twice with DPBS and secondary antibodies diluted in BSA solution were applied for 2 hours at room temperature in the dark. Samples were washed three times with DPBS and Transwell inserts and intestine-on-chip slices were mounted on a glass microscope slide in mounting medium with DAPI (Vector Laboratories #H-2000), while the Matrigel domes containing organoids in Nunc™ Lab-Tek™ Chamber Slide Systems were submerged in the mounting medium. Images were taken using a Leica SP8 CLSM confocal immunofluorescent microscope (10x and 20x objective) and analyzed using the Leica LAS X software. Primary and secondary antibodies are listed in Tables S1 and S2.

Flow cytometry

Organoids were released from Matrigel domes and dissociated using TrypLE Select as described before. Transwell and intestine-on-chip systems were washed twice with DPBS and incubated in TrypLE Select at 37 °C. Every 10 min, detached cells were collected in a microcentrifuge tube, further incubated (5 min, 37 °C) and dissociated to a single cell suspension by gentle repeated pipetting. The single cell suspension was added to a centrifuge tube containing Advanced DMEM/F12 supplemented with FCS (10% vol/vol) and Y-27632 (10 µM) and stored on ice. Previous steps were repeated 3-4 times until the tissue was removed from the Transwell insert and top channel of the intestine-on-chip. The resulting cell suspension was passed through a 70-µm filter, centrifuged (400xg, 5 min, 4 °C) and resuspended in Advanced DMEM/F12 supplemented with FCS and Y-27632. The cells were stained using the Zombie Aqua Fixable Viability Kit (BioLegend #423101) according to the manufacturer’s instructions, fixed in PFA (4% vol/vol DPBS, ThermoFisher #28908) for 10 min and washed with DPBS. Cells were permeabilized and labelled with fluorophore-conjugated antibodies using the BD Perm/Wash buffer (BD Biosciences #554723) according to the manufacturer’s instructions. BD Horizon Brilliant Stain Buffer (BD Biosciences #563794) was included in the antibody mixture in an equal volume to the total volume of the antibodies. Flow cytometry analysis was performed using the Cytek Aurora and the Kaluza Analysis software (Beckman Coulter Life Sciences). Model system-specific unstained, single-antibody stained and fluorescence-minus-one samples were included for the gating strategy and compensation. Antibodies are listed in Table S3.

RNA sequencing and gene expression quantification

Organoids were released from Matrigel domes as described before and lysed in Lysis/Binding Buffer from the mirVana™ miRNA Isolation Kit (ThermoFisher #AM1561). Transwell inserts were washed twice with cold DPBS and cells were lysed in Lysis/Binding Buffer. Cells were

dissociated from the intestine-on-chip as described before, washed twice with DPBS and lysed in Lysis/Binding Buffer. RNA was isolated from cell lysates using the mirVana™ miRNA Isolation Kit (ThermoFisher #AM1561) according to manufacturer's instructions. High RNA integrity (RIN > 8.3) was confirmed using the Agilent RNA ScreenTape (#5067-5576) on the Agilent TapeStation 4200. RNA library preparation and sequencing were performed in one batch at BGI Tech Solutions (Hong Kong) according to the 'DNBSEQ Eukaryotic Strand-specific mRNA library' protocol. Briefly, mRNA was isolated using oligo-dT beads and fragmented. cDNA was synthesized, sequencing adapters were ligated and PCR amplification was performed. Library quality and yield were determined and sequencing was performed using a DNBseq sequencing platform with a 150-bp paired-end (PE150) kit. Adapter sequences and low-quality reads were removed using BGI Tech Solutions' SOAPnuke software (Chen et al., 2018). The trimmed fastQ files were aligned to humanG1Kv37 reference genome (Ensembl Release 75) using Hisat (version 0.1.5) (Kim, Langmead, & Salzberg1, 2015) with default settings, and aligned reads were sorted using SAMtools (version 1.2) (Li et al., 2009). Gene-level quantification was performed using HTSeq-count HTSeq (version 0.6.1p1) (Anders, Pyl, & Huber, 2015) with --mode=union and Ensembl version 75 as gene annotation database. Quality control metrics were calculated for the raw sequencing data using FastQC (version 0.11.3) (Andrews, 2010) and for the aligned reads using Picard-tools (version 1.130). GATK tool SplitNCigarReads was used to split reads into exon segments and hard-clip sequences overhanging into the intronic regions. Variant calling was done using HaplotypeCaller in GVCF mode. All samples were then jointly genotyped by taking the gVCFs produced earlier and running GenotypeGVCFs to create a set of raw SNP and indel calls per chromosome (McKenna, 2010).

Distance matrix analysis

Genes representing the epithelial compartment of the human small intestine were determined using the single-cell RNA sequencing data of the Gut Cell Atlas (Elmentaite et al., 2021, 2020). The data was subsetted to exclude inflammatory bowel disease-related samples (Diagnosis = 'Pediatric Crohn Disease') and select for cells of the small intestine (Region = 'SmallInt'). DEGs were identified using the FindMarkers function of Seurat (version 5.0.2), contrasting the intestinal epithelial cells (category = 'Epithelial') to all other cellular compartments (mesenchymal, neural, endothelial and immune). 'Intestinal epithelial marker genes' were filtered as having adjusted p-value < 0.05 and falling in the fourth quantile of log2FoldChange (corresponding to log2FoldChange ≥ 2.7), resulting in 993 genes. To perform the distance matrix analysis, the Gut Cell Atlas data (Elmentaite et al., 2021, 2020) was subsetted for small intestinal epithelial cells using the same parameters as indicated above and additionally selecting for epithelial cells (category = 'Epithelial') and excluding one donor (Sample.name = 'A32 (411C)') based on the aberrant Paneth cell ratio of 50% from all epithelial cells in an ileal sample. Subsequently, pseudo-bulk data was generated by aggregating counts per age group (variable 'Age_group') and donor (variable 'Sample.name') using the Seurat function AggregateExpresssion. Raw counts of shared genes between the hiPSC-derived intestinal data (organoids, Transwell and intestine-on-chip system) described in this manuscript, the Gut Cell Atlas data and the RNA-sequencing data of the epithelial layer of human duodenal biopsies from healthy adults described by Ramírez-Sánchez et al. (Ramírez-Sánchez et al., 2024) were merged in one count matrix. The euclidean distance between the samples was determined using the VST-normalized expression (DESeq2) of

intestinal epithelial marker genes present in the count matrix using the dist() function in R and scaled per column for the visualization.

Transepithelial electrical resistance in Transwells

Transepithelial electrical resistance (TEER) was measured every second day in Transwell systems with the Millicell ERS-2 Voltohmmeter (Millipore, #MERS00002). For each timepoint, the average resistance from three positions per insert was calculated, and the resistance of an empty insert was subtracted to correct for background. Subsequently, the unit area resistance ($\Omega x cm^2$) was calculated by multiplying the resistance (Ω) by the effective membrane area of the inserts (1.12 cm^2).

Tissue height quantification in intestine-on-chip

Tissue height was calculated from cross-sectional chip slices using Fiji-2 by averaging the shortest and longest distances from the tissue surface in the top channel to the chip membrane and converting to height using the scale bar. For each donor, the average was calculated of four slices from different positions along the length of the chip.

Supplemental references

Anders, S., Pyl, P. T., & Huber, W. (2015). HTSeq-A Python framework to work with high-throughput sequencing data. *Bioinformatics*, *31*(2), 166–169. https://doi.org/10.1093/bioinformatics/btu638

Andrews, S. (2010). FastQC a Quality Control Tool for High Throughput Sequence Data.

Chen, Y., Chen, Y., Shi, C., Huang, Z., Zhang, Y., Li, S., … Chen, Q. (2018). SOAPnuke: A MapReduce acceleration-supported software for integrated quality control and preprocessing of high-throughput sequencing data. *GigaScience*, *7*(1), 1–6. https://doi.org/10.1093/gigascience/gix120

Elmentaite, R., Kumasaka, N., Roberts, K., Fleming, A., Dann, E., King, H. W., … Teichmann, S. A. (2021). Cells of the human intestinal tract mapped across space and time. *Nature*, *597*(7875), 250–255. https://doi.org/10.1038/s41586-021-03852-1

Elmentaite, R., Ross, A. D. B., Roberts, K., James, K. R., Ortmann, D., Gomes, T., … Zilbauer, M. (2020). Single-Cell Sequencing of Developing Human Gut Reveals Transcriptional Links to Childhood Crohn's Disease. *Developmental Cell*, *55*(6), 771-783.e5. https://doi.org/10.1016/j.devcel.2020.11.010

Kim, D., Langmead, B., & Salzberg1, S. L. (2015). HISAT: a fast spliced aligner with low memory requirements Daehwan HHS Public Access. *Nature Methods*, *12*(4), 357–360. https://doi.org/10.1038/nmeth.3317.HISAT

Li, H., Handsaker, B., Wysoker, A., Fennell, T., Ruan, J., Homer, N., … Durbin, R. (2009). The Sequence Alignment/Map format and SAMtools. *Bioinformatics*, *25*(16), 2078–2079. https://doi.org/10.1093/bioinformatics/btp352

McKenna, A. (2010). The Genome Analysis Toolkit: a MapReduce framework for analyzing next-generation DNA sequencing data.

Moerkens, R., Mooiweer, J., Ramírez-Sánchez, A. D., Oelen, R., Franke, L., Wijmenga, C., … Withoff, S. (2024). An iPSC-derived small intestine-on-chip with self-organizing epithelial, mesenchymal and neural cells. *Cell Reports*, *43*(7), 114247. https://doi.org/https://doi.org/10.1016/j.celrep.2024.114247

Ramírez-Sánchez, A. D., Zühlke, S., Aguirre-Gamboa, R., Vochteloo, M., Franke, L., Lundin, K. E. A., … Jonkers, I. H. (2024). Gene expression and eQTL analysis reflect the heterogeneity in the inflammatory status of the duodenal epithelial lining in coeliac disease. *BioRxiv*. https://doi.org/https://doi.org/10.1101/2024.02.29.582756
